# *Halotetraselmis* gen. nov. and *Halotetraselmis indica* comb. nov. (Chlorodendrophyceae, Chlorophyta): New Halophilic Green Algae from Geographically Distant Hypersaline Habitats

**DOI:** 10.3390/microorganisms14102224

**Published:** 2026-10-02

**Authors:** Elena A. Selivanova, Anna D. Temraleeva, Oleg A. Tynnikov, Alexander S. Balkin, Marina E. Ignatenko, Michail M. Yakimov

**Affiliations:** 1 Institute for Cellular and Intracellular Symbiosis of the Ural Branch of Russian Academy of Sciences, Orenburg Federal Research Center of the Ural Branch of Russian Academy of Sciences, 460000 Orenburg, Russia; senior.tynnikov@yandex.ru (O.A.T.); balkinas@yandex.ru (A.S.B.); ignatenko_me@mail.ru (M.E.I.); 2 All-Russian Collection of Microorganisms (VKM), G.K. Skryabin Institute of Biochemistry and Physiology of Microorganisms, Pushchino Scientific Center for Biological Research of the Russian Academy of Sciences, 142290 Pushchino, Russia; temraleeva.anna@gmail.com; 3 Extreme Microbiology, Biotechnology and Astrobiology Group, The Institute of Polar Sciences of the National Research Council (ISP-CNR), 98122 Messina, Italy

**Keywords:** *Halotetraselmis indica*, Chlorodendrophyceae, halophilic microalga, multilocus phylogeny, ITS2 secondary structure, halotolerance

## Abstract

To date, the taxonomic revision of the class Chlorodendrophyceae remains incomplete; the diversity of its halophilic representatives is poorly studied, and the precise phylogenetic position of *Tetraselmis indica*, its only known halophilic species, remains unresolved. Two novel halophilic green microalgal strains related to *Tetraselmis indica*, isolated from geographically distant hypersaline habitats (Crimean lakes and rivers of the Lake Elton basin), were examined using an integrative approach combining morpho-physiological characterization with multilocus molecular phylogenetic analysis (18S rRNA gene, ITS1-5.8S-ITS2 region, 28S rRNA gene, and *rbcL* gene); ITS2 metabarcoding was additionally used to clarify their distribution in the temperate zone of Eurasia. As a result, *Halotetraselmis* gen. nov. and *H. indica* comb. nov. were described. The early-diverging (basal) position of *Halotetraselmis* within Chlorodendrophyceae, the substantial genetic distances separating it from *Tetraselmis*, and its obligate halophily, together with the previously described morphological and chemotaxonomic differences, consistently support recognition of this lineage at the genus level. Genetic markers determined for *H. indica* for the first time (the 28S rRNA gene, the *rbcL* gene, and the ITS1-5.8S-ITS2 region) showed a differing diagnostic value for genus/subgenus-level versus species-level differentiation. Analysis of the ITS1-5.8S-ITS2 region and of ITS2 secondary structure, used for delimitation within *Halotetraselmis*, revealed potentially novel species among unidentified cultured strains. The combination of culture-based methods and metabarcoding yielded new data on the geographic distribution and ecology of the genus. These findings broaden current understanding of the diversity, phylogeny, and ecology of the class Chlorodendrophyceae and represent an important step toward a comprehensive taxonomic revision of the class.

## 1. Introduction

Chlorodendrophyceae is a small class of green algae comprising a single order, Chlorodendrales, a single extant (living) family, Chlorodendraceae, and two genera, *Tetraselmis* and *Scherffelia* [1,2], whereas its other representatives, such as the family Pterosphaeridiaceae, are known exclusively from fossils [3]. The distinct phylogenetic position of Chlorodendrophyceae as one of the two earliest-diverging lineages of the “core” Chlorophyta, has been supported both by molecular phylogenetic data based on 18S rRNA gene analysis [4,5,6], by a number of ultrastructural characters [7,8,9], and subsequently confirmed by chloroplast sequences data [10,11,12], although its precise position relative to Pedinophyceae and the UTC clade (Ulvophyceae, Trebouxiophyceae, and Chlorophyceae) remains unclear and model-dependent even when using large transcriptomic datasets [13].

In green microalgae, genus-level delimitation traditionally relies on a polyphasic approach combining molecular phylogeny, namely significant genetic divergence, with clear morphological, ultrastructural, ecological, physiological, and sometimes chemotaxonomic features, because morphology alone often fails to reflect evolutionary relationships [14,15]. At the same time, there is no universal genetic-distance cutoff for genus-level delimitation in Chlorophyta [16,17]. Within the class Chlorodendrophyceae, which historically comprises only two widely accepted genera, *Tetraselmis* and *Scherffelia* [2,4], the intergeneric boundaries at the 18S rRNA locus are heavily obscured due to high intrageneric variation and poor resolution of the marker [18]. Therefore, a novel well-supported phylogenetic lineage exhibiting genetic distances that substantially exceed this historical baseline can be recognized at the generic level, provided that its status is confirmed by distinct physiological or chemotaxonomic traits.

*Tetraselmis* F.Stein, 1878 is the core genus of Chlorodendrophyceae, comprising 39 species and 2 varieties according to AlgaeBase, most of which have been described based on light and electron microscopy [3,19]. The presence of a single cup-shaped chloroplast with a pyrenoid is the key morphological character distinguishing this genus from *Scherffelia*, which possesses two separate lateral chloroplasts lacking a pyrenoid [20]. The genus *Tetraselmis* is subdivided into four subgenera (*Tetraselmis*, *Prasinocladia*, *Tetrathele*, *Parviselmis*) based on comparative ultrastructural analysis of pyrenoid architecture [21,22,23]. The use of molecular genetic methods has enabled not only the species-level identification of new strains [24,25] but also the description, in recent years, of three new species within the genus: *Tetraselmis indica* [18], *Tetraselmis jejuensis* [26], and *Tetraselmis convallis* [27]. At the same time, many authors noted the incomplete congruence between morphological and molecular genetic data [26,28,29] and emphasized the need to revise the genus using a polyphasic approach including multilocus phylogeny, since 18S data alone are insufficient to clearly delimit extant species or determine their phylogenetic position [18,24]. The complete ribosomal operon, the ITS1 and ITS2 regions, as well as the secondary structure of ITS2, have been shown to be useful for defining internal boundaries within the genus *Tetraselmis* [28,29,30].

Species within *Tetraselmis* thrive in marine, brackish, and freshwater habitats [30], and some are symbionts of heterotrophic protists and metazoans [27,30,31,32,33]. However, only one species, *Tetraselmis indica*, isolated from Goa salterns (India), has been described as growing in an unusually broad range of salinities, namely from marine salinity (~35 psu) to saturation (up to 350 psu) [22]. The species was described using a polyphasic approach, but its precise placement within the *Tetraselmis* clade could not be resolved based on 18S rRNA gene phylogenetic analysis alone, as *T. indica* exhibits a large genetic distance from other described members of the genus [18]. Despite this uncertain phylogenetic position, the placement of *T. indica* within the *Tetraselmis* clade has not been revised since its original description, although multigene arrays (18S + ITS + 28S + *rbcL*) are now available for other lineages within the genus [25,27].

Subsequent research on *Tetraselmis indica* has focused on its physiology and biotechnological potential, including wastewater phytoremediation and biodiesel feedstock production [34], survival strategies in darkness via resting cells [35], microalgae–bacteria associations and symbiotic consortia [36,37], lipids and photosynthetic pigments content under mixotrophic cultivation [38], and the development of cultivation systems for biomass production [39]. Notably, physiological studies have also documented asymmetric cell division in *Tetraselmis indica*, which is atypical for the genus [40] and the absence of loroxanthin, a characteristic pigment of the genus *Tetraselmis* [41]. These documented physiological and biochemical differences further highlight the need to clarify the relationships between this taxon and other members of the genus. In addition to *Tetraselmis indica*, other halophilic *Tetraselmis* spp. have been described from natural hypersaline lakes in Western Australia [42], from coastal waters of Korea (80 PSU) [43], from a hypersaline pond near Great Salt Lake, Utah, USA [44], and from saline rivers flowing into Lake Elton [36]. However, the species identity of these halophilic microalgal strains remains unresolved. This further underscores how little is known about halophilic representatives of Chlorodendrophyceae, including the possibility that there are species among them that have not yet been validly described. In the original description of *Tetraselmis indica*, its geographic distribution was characterized as disjunct, represented by India (Goa salterns) and North America (Great Salt Lake) [18], likely reflecting insufficient sampling at the time of the study. Previously, we detected phylotypes closely related to *Tetraselmis indica*, using 18S metabarcoding of the V4 region, in hypersaline water bodies of the temperate Eurasia, namely in Crimea and in salty rivers inflowing Lake Elton [45,46]. These findings indicate a more widespread distribution of halophilic representatives of the class Chlorodendrophyceae in hypersaline environments. However, despite its widespread use, 18S metabarcoding has limited resolution at the species level, whereas ITS2 metabarcoding—a method generally considered more accurate for distinguishing individual species [47]—cannot currently be used to detect *Tetraselmis indica* in environmental samples due to the absence of reference sequences for this species in the GenBank database.

Taken together, this brief review demonstrates the relevance and need for further research on both *Tetraselmis indica* and other halophilic representatives of the genus *Tetraselmis*, without which our current understanding of Chlorodendrophyceae remains incomplete. Accordingly, the aim of this study is to characterize novel halophilic green algal strains related to *Tetraselmis indica*, isolated from geographically distant hypersaline habitats (saline lakes in Crimea and rivers of the Lake Elton basin), using an integrative polyphasic approach combining morpho-physiological characterization with multilocus molecular phylogenetic analysis (18S rRNA gene, ITS1-5.8S-ITS2 region, 28S rRNA gene, and *rbcL* gene), and to clarify its distribution across water bodies of the temperate zone of Eurasia based on ITS2 metabarcoding data.

## 2. Materials and Methods

### 2.1. Culture Isolation

The microalgal strain ICIS64 was isolated from a brine sample collected in August 2021 from a saltern near Sivash Bay, Crimea (45°19′43″ N, 35°24′01″ E). Brine salinity at the sampling site was 160 g/L, with a pH of 7.46. The second microalgal strain ICIS65 was isolated from a brine sample collected in 2018 from the hypersaline Malaya Smorogda River (49°05′46″ N, 46°43′59″ E), which flows into Lake Elton (Volgograd Region, Russia). Brine salinity at the sampling site was 110 g/L, with a pH of 7.88. Both water bodies were shallow (depth ≤ 20 cm at the time of sampling), precluding depth-stratified sampling; plankton samples were therefore collected as integrative samples encompassing the surface, water column, and near-bottom layers. Algologically pure cultures were obtained by direct isolation of single cells (resting cells, in the case of strain ICIS64) under an inverted Nikon TS2 microscope (Nikon, Tokyo, Japan) using Pasteur pipettes with drawn-out tips, as previously described [36].

### 2.2. Culture Medium and Conditions

At the initial stages of work, cultures were maintained in a modified synthetic mineral medium OPS (medium salinity 100‰), containing 82.6 g NaCl, 8.3 g MgSO_4_, 2.5 g KNO_3_, 0.2 g K_2_HPO_4_, and 1 g NaHCO_3_ per liter of distilled water, supplemented with solutions of trace elements and vitamins, prepared according to Guillard’s (F/2) Marine Enrichment medium [48]. Subsequently, following determination of the optimal salinity range, the salt composition of the culture medium was adjusted by reducing NaCl to 60 g/L, MgSO_4_ to 6 g/L, and NaHCO_3_ to 0.5 g/L, lowering the overall medium salinity to 70‰. Cultures were incubated in a light incubator KS-200 (Evrolab, Saint Petersburg, Russia) under a 12 h light/12 h dark photoperiod at an irradiance of 5000 lx provided by cool white fluorescent lamps and at a temperature of 25 °C.

The studied strains were deposited in the Network Collection of Symbiotic Microorganisms and Their Consortia at the Institute of Cell and Intracellular Symbiosis, Ural Branch of the Russian Academy of Sciences as ICIS64 and ICIS65, as well as in the All-Russian Collection of Microorganisms (VKM Al-571 and VKM Al-570, respectively).

### 2.3. Study of Morphological Features

Morphological features of the microalgae were examined using phase-contrast microscopy with oil immersion (Axioscope A1 microscope; Carl Zeiss, Oberkochen, Germany); photographic documentation was performed using an AxioCam 105 color digital camera (Carl Zeiss, Oberkochen, Germany) with ZEN 2.3 (blue edition) software (Carl Zeiss, Oberkochen, Germany). Morphometric measurements were performed on two independent cultures (strains ICIS64 and ICIS65) grown under optimal salinity conditions; samples were collected twice, after 2 and 8 weeks of cultivation, allowing for the measurement of both motile and non-motile (resting) cells (with the latter predominating in the older cultures).

For scanning electron microscopy (SEM), cells of both strains, grown in liquid mineral medium (salinity 70 g/L) to the late exponential growth phase, were pelleted by centrifugation at 5000 rpm (2292× *g*) for 15 min (Centrifuge 5418R, Eppendorf, Hamburg, Germany). The cell pellet was fixed with 2.0% glutaraldehyde (*v*/*v*, final concentration) diluted in NaCl solution (final salinity 70 g/L) for 1 h. The fixative was removed by washing twice with NaCl solution (salinity 35 g/L). The fixed material was placed onto poly-L-lysine-coated coverslips for 10 min to allow cells to adhere to the poly-L-lysine coating. Dehydration was achieved through a graded ethanol series (25, 50, 75, and 90% (*v*/*v*) ethanol) followed by pure ethanol (twice), 10 min per step at room temperature. Samples were then subjected to critical-point drying with liquid CO_2_ (Quorum K850 Critical Point Dryer, Quorum Technologies Ltd., London, UK). Dried samples were sputter-coated with a gold film (Quorum Q150R S Plus, Quorum Technologies Ltd., London, UK) prior to examination using a TESCAN Mira 3 scanning electron microscope (Tescan Brno s.r.o., Brno, Czech Republic) at an accelerating voltage of 5 kV.

### 2.4. DNA Extraction, PCR, and Sequencing

DNA was extracted from microalgal cultures using the HiPure HP Plant DNA Kit (Magen Biotechnology Co., Ltd., Guangzhou, China) according to the manufacturer’s instructions. Nucleotide sequences of the 18S rRNA, 28S rRNA and *rbcL* genes, as well as the ITS1-5.8S-ITS2 region, were amplified using 2× Phanta Flash Master Mix (Dye Plus) (Vazyme, Nanjing, China). Primers and amplification conditions are listed in Table 1. Amplification was performed using a T100 thermal cycler (Bio-Rad, Hercules, CA, USA). Target PCR products were visualized by electrophoresis in a 1% agarose gel. Amplicons were purified from the agarose gel using the SKYGel Purification Kit EGC219 L (TIANGEN Biotech, Beijing, China). DNA concentration was measured using a Qubit 4 fluorometer with the Qubit™ 1X dsDNA HS Assay Kit (Invitrogen, Carlsbad, CA, USA).

Sequencing of the purified PCR products was performed by Evrogen (Moscow, Russia). Assembly of the marker gene sequences from the sequencing reads was carried out using MEGA11 software.

### 2.5. Phylogenetic Analysis

The nucleotide sequences obtained for strain ICIS64 were deposited in the GenBank database under the following accession numbers: PZ477976 (18S rRNA gene), PZ639060 (28S rRNA gene), PZ485853 (ITS1-5.8S-ITS2), and PZ495605 (*rbcL*); for strain ICIS65, under accession numbers PZ477977, PZ639059, PZ485854, and PZ495606, respectively. Homologous sequences were retrieved from the GenBank database using the BLASTn algorithm (https://blast.ncbi.nlm.nih.gov (accessed on 3 July 2026)) [53]. Taxon names were assigned in accordance with the international electronic database AlgaeBase (https://www.algaebase.org (accessed on 3 August 2026)). [3]. Details of all sequences (taxon names, strain designations, and GenBank accession numbers) used in the phylogenetic analyses are provided in Table S1. It should be noted that the class Chlorodendrophyceae is a small evolutionary lineage comprising only two generally accepted extant genera. The sequence datasets assembled for the 28S rRNA and *rbcL* genes include all authentic, high-quality sequences available in public repositories to date. While these datasets are numerically limited by the current completeness of global databases, they comprehensively represent the known taxonomic diversity of this class and closely related outgroups, thereby ensuring that the phylogenetic distinctness of the proposed taxon is not artificially inflated by insufficient sampling. Multiple sequence alignment was performed in MEGA11 using the ClustalW algorithm [54].

Evolutionary analyses were conducted in MEGA11 [54]. The best-fit substitution model for each dataset was selected using the Model Selection tool in MEGA11. Evolutionary divergence between 52 nucleotide 18S rDNA sequences (1690 bp) was estimated using the Kimura 2-parameter model with gamma-distributed rate variation and a proportion of invariable sites (Kimura-2 (G + I)) [55]. Genetic distances between 24 nucleotide sequences of the 28S rRNA dataset (1379 bp) were calculated using the Tamura-Nei model with gamma-distributed rate variation among sites (TN93 + G) [56].

Maximum likelihood (ML) phylogenetic analysis was performed using IQ-TREE version 3.0.1 [57], with 1000 replicates for both the ultrafast bootstrap and the SH-aLRT branch test. The best-fitting substitution model was determined with the same software using the Bayesian Information Criterion (BIC). For the 52 18S rRNA gene nucleotide sequences (1690 bp) the TN + F + R3 model was selected. For the 28S rRNA gene, the same approach was applied, but 24 sequences (1379 bp) were aligned, and the TN + F + R2 model was selected.

To construct the concatenated phylogeny of Chlorodendrophyceae, we used the untrimmed alignments of 18S rDNA (34 sequences) and 28S rRNA (22 sequences) prepared previously [27]. One 18S rRNA gene sequence of *Tetraselmis* sp. DS3 (KP100528) was excluded owing to its short length (1035 bp). Overhanging base pairs were manually trimmed after adding our own sequences, resulting in final alignments of 1610 bp and 1379 bp for 18S and 28S, respectively. The two single-gene alignments were concatenated to produce a dataset of 36 sequences totaling 2989 bp, including 6 outgroup sequences. For the concatenated 18S-28S dataset, the TN + F + R2 model was again selected. The *rbcL* gene alignment comprised 16 sequences (1237 bp), for which the GTR + F + I model was selected.

In addition to the ML analyses, Bayesian inference was performed using MrBayes 3.2.7a [58]. Analyses were run under a substitution model with two substitution types (nst = 2) and gamma-distributed rate variation across sites with four rate categories (rates = gamma, ngammacat = 4). Each run comprised 5,000,000 generations, with sampling every 1000 generations; the first 1250 trees (25%) were discarded as burn-in. The resulting consensus trees were used to extract posterior probability support values.

The resulting tree was visualized, edited, and annotated using FigTree v1.4.4 (http://tree.bio.ed.ac.uk/). For comparison of tree topologies among the analyzed sequences, we used data from the publications [18,26,27].

### 2.6. Folding of ITS2

ITS2 secondary structure folding and compensatory base change (CBC) analyses were performed to clarify the phylogenetic and taxonomic status of the new strains relative to their closest homologs. To define the boundaries of the newly obtained ITS2 sequences of the new strains and their closest homologs, raw sequences were annotated and trimmed using the annotation tool implemented in the ITS2 Database (http://its2.bioapps.biozentrum.uni-wuerzburg.de/ (accessed on 28 July 2026)) [59,60]. ITS2 secondary structures were folded using the RNAfold web server (http://rna.tbi.univie.ac.at/cgi-bin/RNAWebSuite/RNAfold.cgi (accessed on 31 July 2026)) based on the minimum free energy principle. A multiple sequence–structure alignment was generated in 4SALE v1.7 [61,62], and the resulting secondary structures were screened for compensatory base changes (CBCs) using 4SALE, following the criteria of Coleman [63,64] and Müller et al. [65]. The obtained secondary structure was compared with the previously reported ITS2 secondary structure of *Tetraselmis* [30]. Visualization of the predicted ITS2 secondary structures was performed using PseudoViewer (compact version 3.0) [66].

### 2.7. Halotolerance Assessment

Both strains were examined for their ability to survive and proliferate as a function of the salinity of the culture medium. The range of halotolerance was assessed across salinities from 2.5 to 250 g/L. Modified OPS media with salinities of 2.5, 35, 50, 65, 75, 100, 125, 150, 165, 185, 200, 225, and 250 g/L were prepared by proportionally adjusting the NaCl and MgSO_4_ contents. The resulting salinity of each medium was verified using a Master S-28α portable refractometer (Atago, Tokyo, Japan). Microalgae cultivation was performed in triplicate using sterile 24-well plates (NEST, Wuxi, China) at 25 °C in a light incubator. The initial inoculum concentration for both cultures, ICIS64 and ICIS65, was approximately 11,000 cells/mL. This value was determined by counting cells in a Nageotte chamber (Marienfeld, Lauda-Königshofen, Germany). A 2 µL aliquot of this inoculum was added to each well containing 2 mL of mineral medium of the appropriate salinity, resulting in an initial concentration of approximately 11 cells/mL per well. Cell counts were performed unhypersalinityder an inverted Nikon TS2 microscope (Nikon, Tokyo, Japan) in 10 fields of view across the full depth of the well (100× magnification). Growth was scored using a semi-quantitative scale based on the total number of cells observed across all 10 fields: 1 point for 1–10 cells, 2 points for more than 10 but fewer than 100 cells, and 3 points for more than 100 cells. The experiment was conducted in triplicate and monitored for 24 and 26 days for strains ICIS64 and ICIS65, respectively. Cell counts for strain ICIS64 were performed on days 2, 4, 7, 10, 14, and 24, and for strain ICIS65 on days 2, 6, 12, 14, 16, 20, and 26. If no cells were observed in 10 examined fields, the entire well was scanned to confirm the absence of viable cells. In addition, cell morphology was assessed using light microscopy, and representative images were captured during observation (as described above). Results were visualized as a heatmap using Excel.

### 2.8. Analysis of Distribution Based on ITS2 Metabarcoding

A total of 61 environmental samples were sequenced from hypersaline water bodies located in different geographical regions (Crimean Peninsula, Kulunda Steppe, and Volgograd and Orenburg regions of Russia). Sampling and DNA extraction protocols were as described previously [45]. DNA libraries were prepared following the two-step Illumina protocol, using primers carrying sequencing overhangs at their 5′ ends. Libraries for the ITS2 barcode were generated using the primers ITS3 (GCATCGATGAAGAACGCAGC) and ITS4 (TCCTCCGCTTATTGATATGC) [51]. Library quality was assessed by horizontal agarose gel electrophoresis. Amplicons were then purified by solid-phase reversible immobilization using AMPure XP paramagnetic beads (Beckman Coulter, Brea, CA, USA), following Illumina’s recommendations. Paired-end sequencing (2 × 300 bp) was performed on the Salus Pro platform (Salus Biomed, Shenzhen, China) using the Salus sequencing reagent kit (PRM-PE300-80M). Raw read quality was assessed using FastQC v.0.12.1. Primer sequences used for amplification were removed using Cutadapt (version 4.1) [67]. Further processing was performed in R 4.4.0 using the DADA2 package (version 1.32.0), including quality filtering of reads (maximum expected errors = 2), estimation of sequencing error rates, removal of chimeric sequences, and generation of amplicon sequence variants (ASVs). The resulting ASVs were filtered by minimum (200 bp) and maximum (270 bp) length.

To assign the taxonomy of ASVs, the naive Bayesian classifier was used against the UNITE database (Version 10.0) [68] supplemented with 27 Chlorophyta sequences from the Laboratory Collection (Network Collection of Symbiotic Microorganisms and Their Consortia at the Institute of Cellular and Intracellular Symbiosis). The classifier was run with a minimum bootstrap confidence threshold of 0.8 (80%) for genus-level assignments and 0.9 (90%) for species-level assignments. To identify ASVs homologous to the ITS2 sequences obtained from the cultured microalgae, we applied the BLASTn search with a minimum identity threshold of 99% and a query coverage threshold of 100% over the ITS2 region, retaining only those ASVs that matched the top hit with at least 99% sequence similarity to avoid ambiguous assignments.

Raw sequence data have been deposited in the NCBI Sequence Read Archive under BioProject accession PRJNA1475234 (Biosample accessions SAMN60634155–SAMN60634214).

## 3. Results

### 3.1. Morphological Characteristics of Microalgal Strains ICIS64 and ICIS65

**Figure 1:**
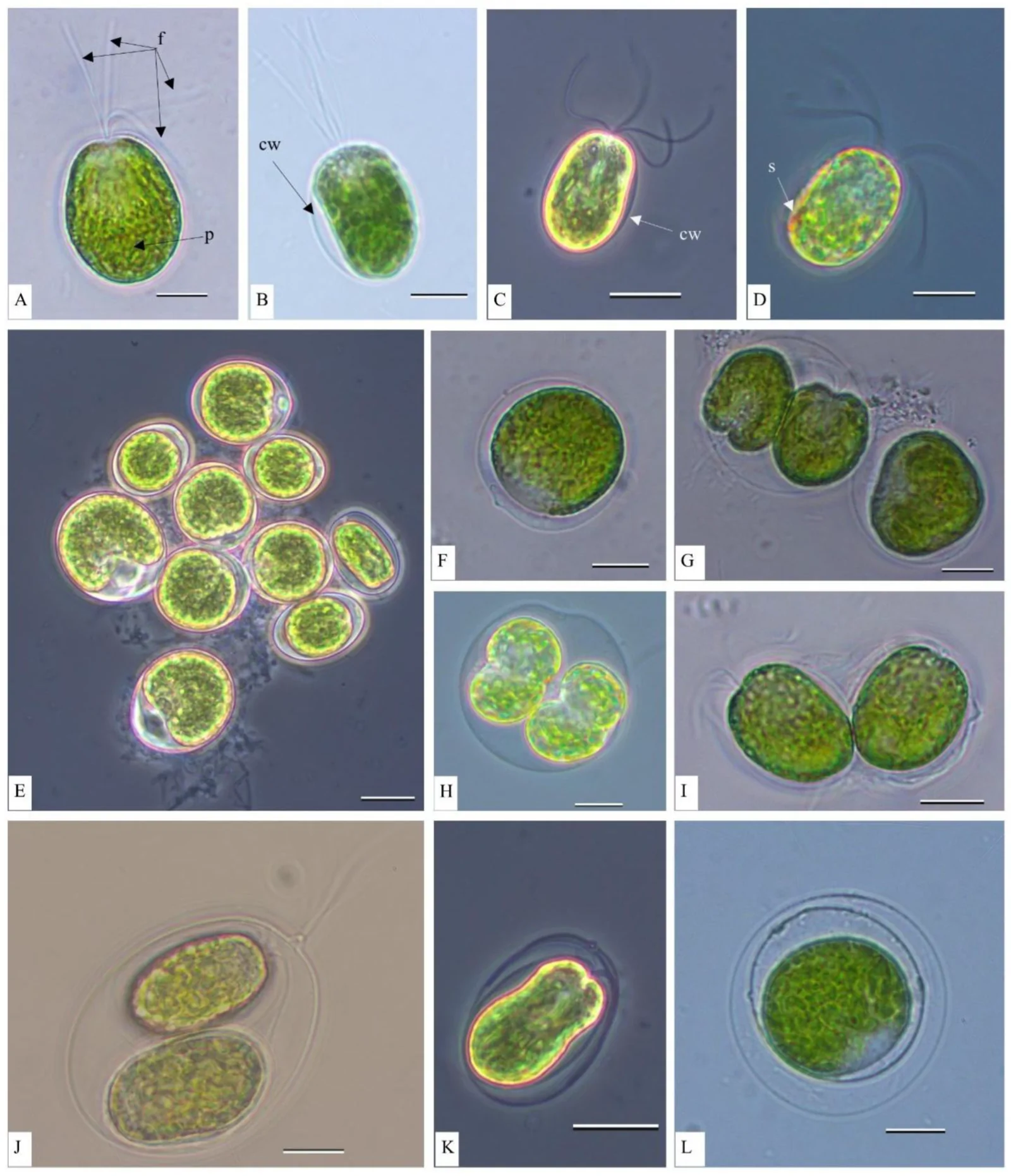
Light micrographs of cells of strains ICIS64 (**A**,**B**,**F**,**G**,**I**,**L**) and ICIS65 (**C**,**D**,**E**,**H**,**J**,**K**). Flagellate (motile) stage: (**A**–**D**), arrows indicate the flagella (f), pyrenoid (p), cell wall (cw), and stigma (s). (**E**,**F**) Vegetative (non-motile) stage. (**G**–**J**) Cell division within the wall of the mother cell, giving rise to two (**G**,**I**,**J**) or four (**H**) daughter cells. Formation of resting cells beneath a thickened, two-layered wall (**K**,**L**). Scale bar—10 μm.

Light microscopy observations showed that both strains, ICIS64 and ICIS65, shared similar morphological features and were preliminarily assigned to the genus *Tetraselmis*. This assignment was based on several key morphological characters: the presence of an invagination at the anterior end and four equal flagella arranged in two opposing pairs (Figure 1A,D). Cells were slightly compressed, elliptical in outline when viewed from the narrow side (Figure 1B–D), and ovoid, with a slightly broadened posterior end, when viewed from the broad side (Figure 1A,G). Cells of strain ICIS65 were 18.5–27.3 μm long (mean 22.2 ± 0.2 μm, *n* = 77), 16.4–24.7 μm wide (mean 20.2 ± 0.3 μm, *n* = 54), and 14.0–18.1 μm thick (mean 15.9 ± 0.2 μm, *n* = 22). Cells of strain ICIS64 were slightly more elongated: at a comparable length of 17.4–28.7 μm (mean 22.1 ± 0.2 μm, *n* = 96), they were 15.6–25.0 μm wide (mean 19.2 ± 0.2 μm, *n* = 69) and 10.9–18.2 μm thick (mean 14.8 ± 0.3 μm, *n* = 27).

Cells contained a single cup-shaped chloroplast, ranging in color from dark green to yellowish-green, with a large, conspicuous pyrenoid (Figure 1A). A red-orange stigma was located in the posterior third of the cell (Figure 1D). In addition to the motile stage, the cell cycle also included a non-motile stage, which predominated in older cultures. Non-motile cells became rounded, with the theca separated from the protoplast by a gap (Figure 1E,F,L). In strain ICIS64, protoplast diameter was 15.8–24.3 µm (mean 20.5 ± 0.3 µm, *n* = 51), and theca length and width were 22.3–42.0 µm (mean 34.3 ± 0.7 µm) and 17.2–38.2 µm (mean 30.5 ± 0.6 µm), respectively (*n* = 51). In strain ICIS65, protoplast diameter was 13.5–27.8 µm (mean 20.0 ± 0.3 µm, *n* = 92), and theca length and width were 22.7–48.1 µm (mean 30.6 ± 0.6 µm) and 14.8–41.9 µm (mean 26.0 ± 0.5 µm), respectively (*n* = 92). Asexual reproduction occurred by division within the parent theca, producing 2 or 4 cells (Figure 1G,H,I); this most commonly took place during the non-motile stage, although flagella were occasionally observed to persist on the parent theca (Figure 1J). In daughter cells, flagella developed while the cells were still within the parent theca (Figure 1G,J). One of the daughter cells was also observed to invert within the parental periplast, after which the two daughter cells came to lie in opposite orientations (Figure 1G). The strains were also noted to form resting cells with thickened cell walls, sometimes doubled owing to retention of the old cell wall (Figure 1E,K,L).

Compared with *Tetraselmis indica*, the only previously described representative of the genus isolated from a hypersaline water body [18], the strains examined in this study showed similar morphological features at the light-microscopic level, including the position of the stigma, although they were larger in size than the authentic strain (10–25 × 7–20 × 6.5–18 μm), albeit with overlapping size ranges. This difference may be attributable to the fact that the authentic strain has been maintained in culture at marine salinity rather than at its optimal salinity (approximately 70%).

**Figure 2:**
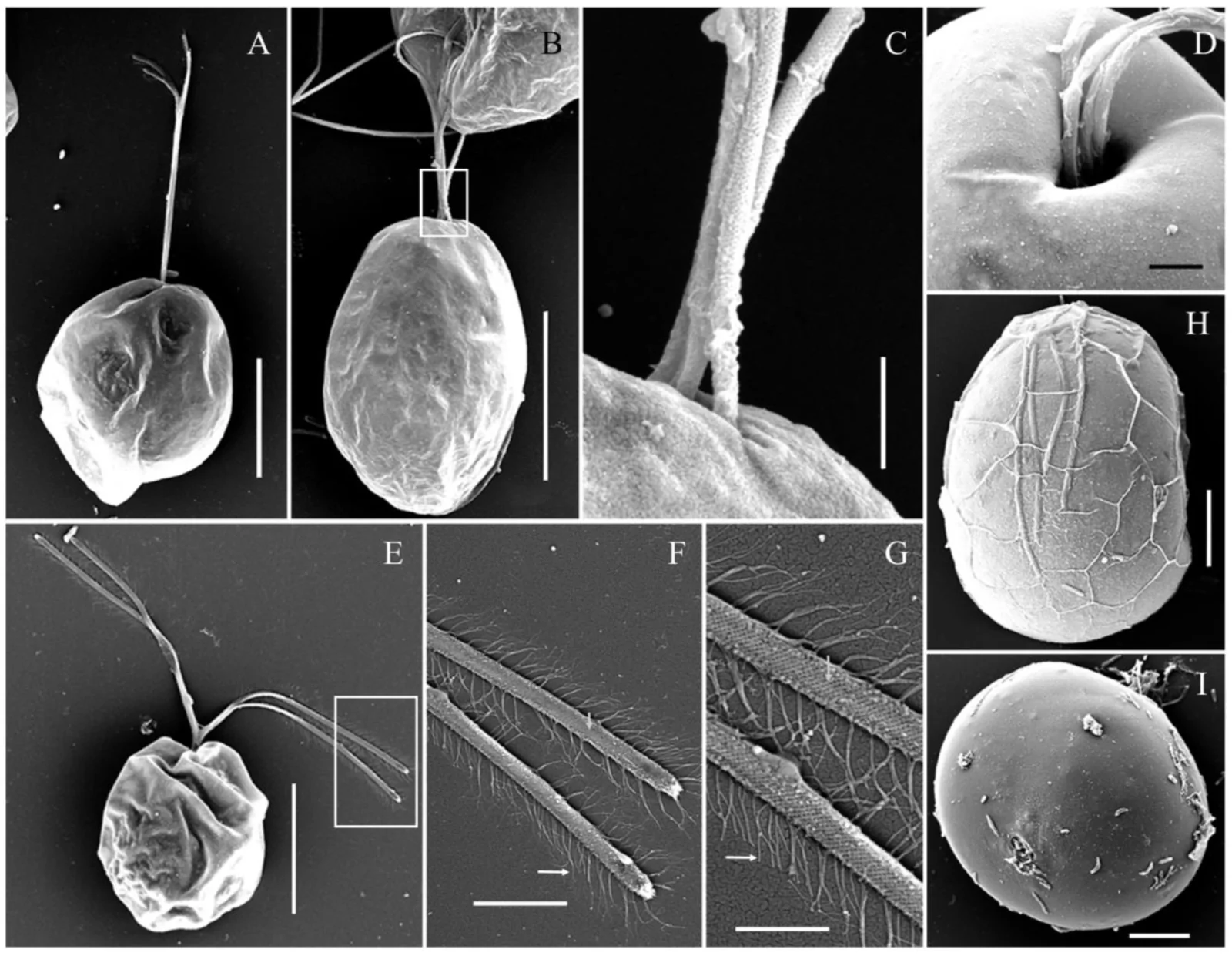
Micrographs of strain ICIS64 cells obtained using scanning electron microscopy. (**A**,**B**) lateral views of cells showing the depression on the apical side; (**C**) lateral and (**D**) apical views of the emerging flagella; (**E**) paired arrangement of the flagella and their general appearance; (**F**,**G**) magnified micrographs of the flagella covered with scales (grey arrows) and thin hairs (white arrows); (**H**) flagellate cell covered externally by the theca; (**I**) resting cell with a dense wall and bacteria attached to its surface (the apical papilla, marking the former site of flagellar attachment, is visible). Scale bars: (**A**,**B**,**E**) 10 μm; (**H**,**I**) 5 μm; (**F**) 2 μm; (**C**,**D**,**G**) 1 μm.

SEM micrographs of cells of the both strains showed that the outer theca layer did not adhere tightly to the cell and formed folds as the protoplast shrank during sample preparation (Figure 2A,E,H and Figure S1A,F). Well-preserved cells, however, were ovoid in shape and displayed a characteristic invagination at the anterior end, from which four flagella emerged (Figure 2B and Figure S1B). The flagella emerged from a slit at the center (in the depth) of the apical depression at the anterior part of the cell, with two distinct creases in the apical region of the cell body (Figure 2A,C,D and Figure S1A–D); the invagination, however, did not extend onto the lateral surface. The four flagella emerged together and were arranged in two opposite pairs at the apical pole (Figure 2B,C,E and Figure S1F). Magnified SEM views of the flagella revealed that they were covered along their entire length by numerous fine hairs approximately 1.0–1.5 μm long, as well as by scales (Figure 2F,G and Figure S1G,H). The flagellar scales in the surface layer were arranged in regular, orderly rows, tightly abutting one another diagonally and covering the flagellum along its full length, thereby producing a characteristic rhomboid pattern on the flagellar surface (Figure 2G and Figure S1H). The flagellar tip tapered abruptly, ending in a slightly rounded apex (neither acute nor bluntly truncated) (Figure 2F and Figure S1G,H). Resting cells were represented both by flagellate cells covered externally by the theca and by non-flagellate cells with a dense wall, possibly representing successive stages in the transition to the resting state (Figure 2H,I and Figure S1E). Bacterial attachment was observed on the surface of resting cells as well as on the flagella of vegetative cells (Figure 2E,H,I and Figure S1C,D,F,G).

### 3.2. Phylogenetic Analysis

#### 3.2.1. 18S rRNA Gene Phylogeny

**Figure 3:**
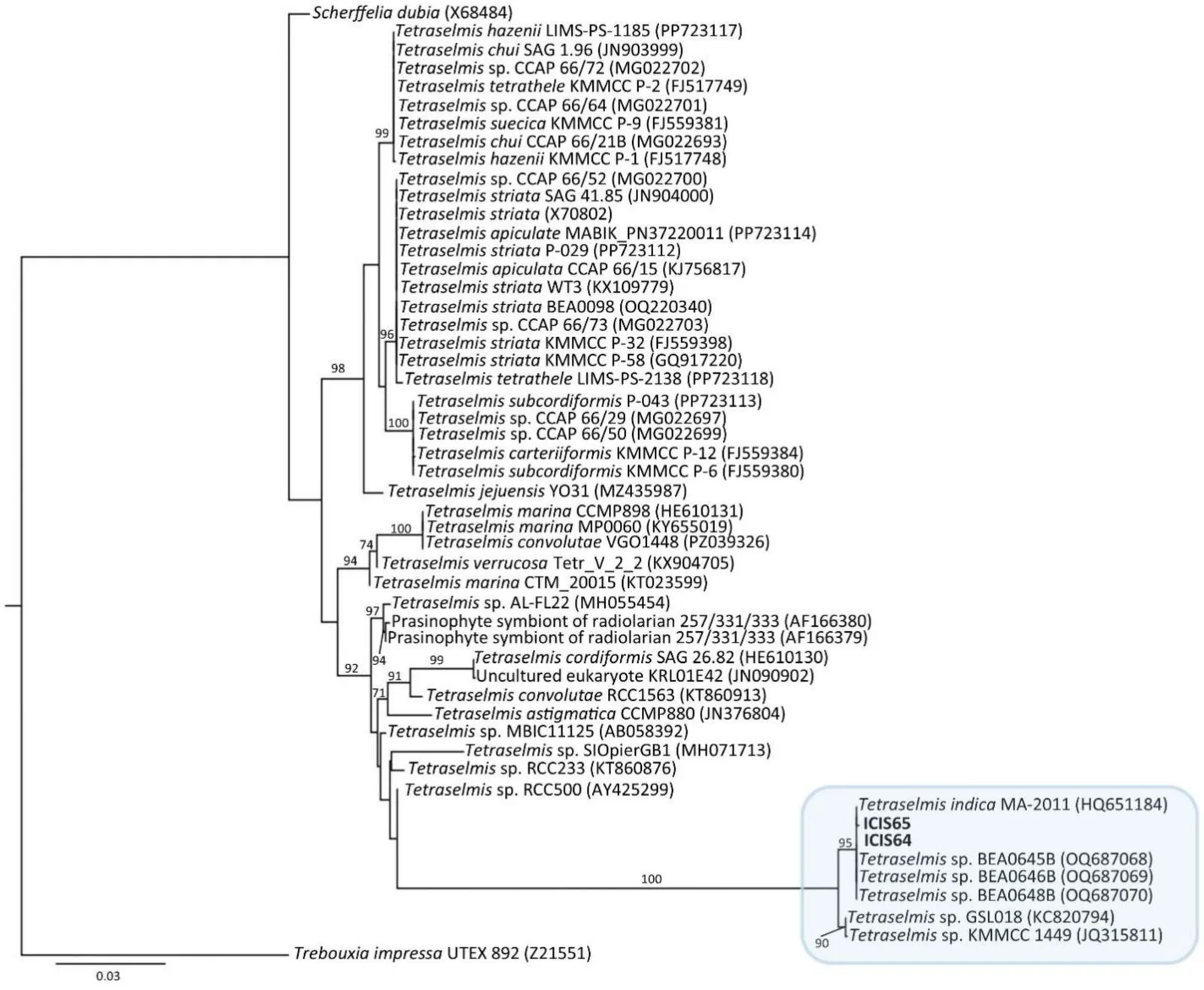
Maximum likelihood tree of Chlorodendrophyceae showing the phylogenetic position of the new strains ICIS64 and ICIS65, constructed based on comparison of 18S rDNA nucleotide sequences (1690 bp). The TN + F + R3 model was selected as the optimal evolutionary model. Statistical support for tree nodes is shown as bootstrap (BP) values; BP values ≤ 70% are not shown. The nucleotide sequence of *Trebouxia impressa* UTEX 892 was used as the outgroup.

The 18S rRNA gene nucleotide sequences of strains ICIS64 and ICIS65 were found to be similar. A BLAST search showed that the closest homologs for the analyzed sequences were the nucleotide sequences OQ687068 and OQ687070, belonging to the cultures *Tetraselmis* sp. BEA0645B and BEA0648B, respectively, isolated from a coastal lagoon in the south of Gran Canaria Island (Canary Islands, Spain). Alignment analysis of 18S rDNA sequences of representatives of the class Chlorodendrophyceae showed that the new strains were placed within the genus *Tetraselmis*, although with very low support (<70%) (Figure 3). Phylogenetic analysis of the 18S rRNA gene sequences showed that the new strains, together with the undescribed strains of their closest homologs, formed a single clade *Tetraselmis indica* (Figure 3), clustering with the authentic strain *Tetraselmis indica* MA-2011. As previously described [18], the *Tetraselmis indica* clade forms a separate, long branch within the tree of the genus *Tetraselmis*, albeit with low statistical support. In addition to the *Tetraselmis indica* clade, this branch also contains a cluster formed by the nucleotide sequences of the culture *Tetraselmis* sp. GSL018 (KC820794), isolated from an evaporation pond with a salinity of 6.0%, located near the Great Salt Lake (Utah, USA) [44], and the culture *Tetraselmis* sp. KMMCC 1449, isolated from seawater (Korea). However, when a maximum likelihood analysis was made using the MEGA11 program and another optimal model Kimura-2 (G + I), the position of the *Tetraselmis indica* clade shifted to become external relative to the other representatives of *Tetraselmis*, with high statistical support (Figure S2), as was also shown in the study by Hyung et al. (2021) [26]. Thus, using the 18S gene as a genetic marker, it is not possible to precisely determine the phylogenetic position of the branch into which the new strains ICIS64 and ICIS65 fell.

**Figure 4:**
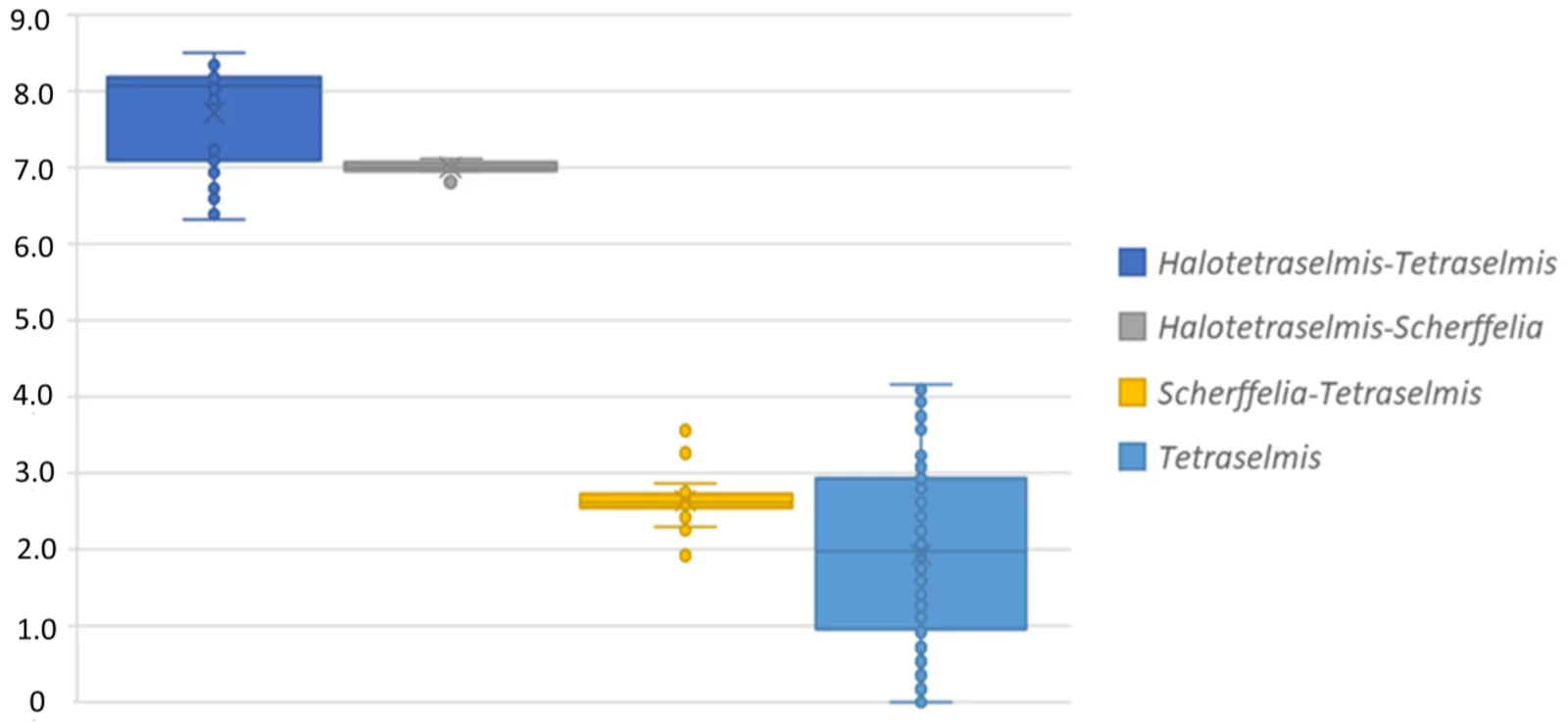
18S rRNA genetic distances (%) among genera within the Chlorodendrophyceae and intrageneric genetic distances of *Tetraselmis*. The borders of the box show the first and third quartiles, the horizontal line is the median value, the ‘whiskers’—the spread.

The genetic distance between the nucleotide sequences of strains ICIS64 and ICIS65 and the closest sequences of *Tetraselmis* sp. GSL018 (KC820794) and *Tetraselmis* sp. KMMCC 1449 (JQ315811) was 0.65–0.79% (Table S2), whereas the genetic distances to other representatives of the genus *Tetraselmis* ranged from 6.33 to 8.51%, considerably higher than the level of divergence between other strains within the genus *Tetraselmis* (excluding those listed above), which did not exceed 4.16% (Figure 4). Notably, the genetic distances at the 18S gene between representatives of the genus *Tetraselmis* and *Scherffelia dubia*, a representative of another genus within the class Chlorodendrophyceae, ranged from 1.93 to 3.63%, whereas for representatives of the *Tetraselmis indica* clade, these distances were 6.82–7.11%.

Thus, the genetic distance values of the *Tetraselmis indica* clade considerably exceeded the level of intrageneric distances characteristic of the genus *Tetraselmis*, which led us to hypothesize that the new strains, together with the previously described strain *Tetraselmis indica* MA-2011, as well as the unidentified cultures *Tetraselmis* sp. GSL018 and *Tetraselmis* sp. KMMCC 1449, represent a separate genus within the class Chlorodendrophyceae, which we named *Halotetraselmis*, since most of the strains had been isolated from hypersaline conditions and exhibit halophilic behavior. To test this hypothesis and to clarify the phylogenetic position of the isolated strains, additional genetic markers were sequenced: the 28S rRNA and *rbcL* genes.

#### 3.2.2. 28S rRNA Gene and Concatenated 18S rRNA-28S rRNA Gene Phylogeny

The two partial sequences of the 28S rRNA gene (1368 bp and 1360 bp, respectively) were identical for strains ICIS64 and ICIS65 and represent the first 28S rRNA data reported for *Halotetraselmis*. Sequence similarity to *Tetraselmis* representatives available in GenBank did not exceed 86.71%. Genetic distances between the new strains and most *Tetraselmis* species ranged from 14.9% to 17.2% (Table S3), with a notably higher distance of 27.7% from *Tetraselmis convolutae* strain C23181 (PV505452). The genetic distance from *Scherffelia dubia*, representing a distinct genus within Chlorodendrophyceae, was 15.3%. For comparison, the intergeneric distance between *Scherffelia dubia* and most *Tetraselmis* species ranged from 4.4% to 8.4% (11.1% with *T. convolutae*)—approximately half the magnitude of the distances separating *Halotetraselmis* from both genera. This indicates that the genus *Halotetraselmis* is substantially more divergent from both *Tetraselmis* and *Scherffelia* than these two established genera are from each other, a pattern consistent with differentiation at genus level.

**Figure 5:**
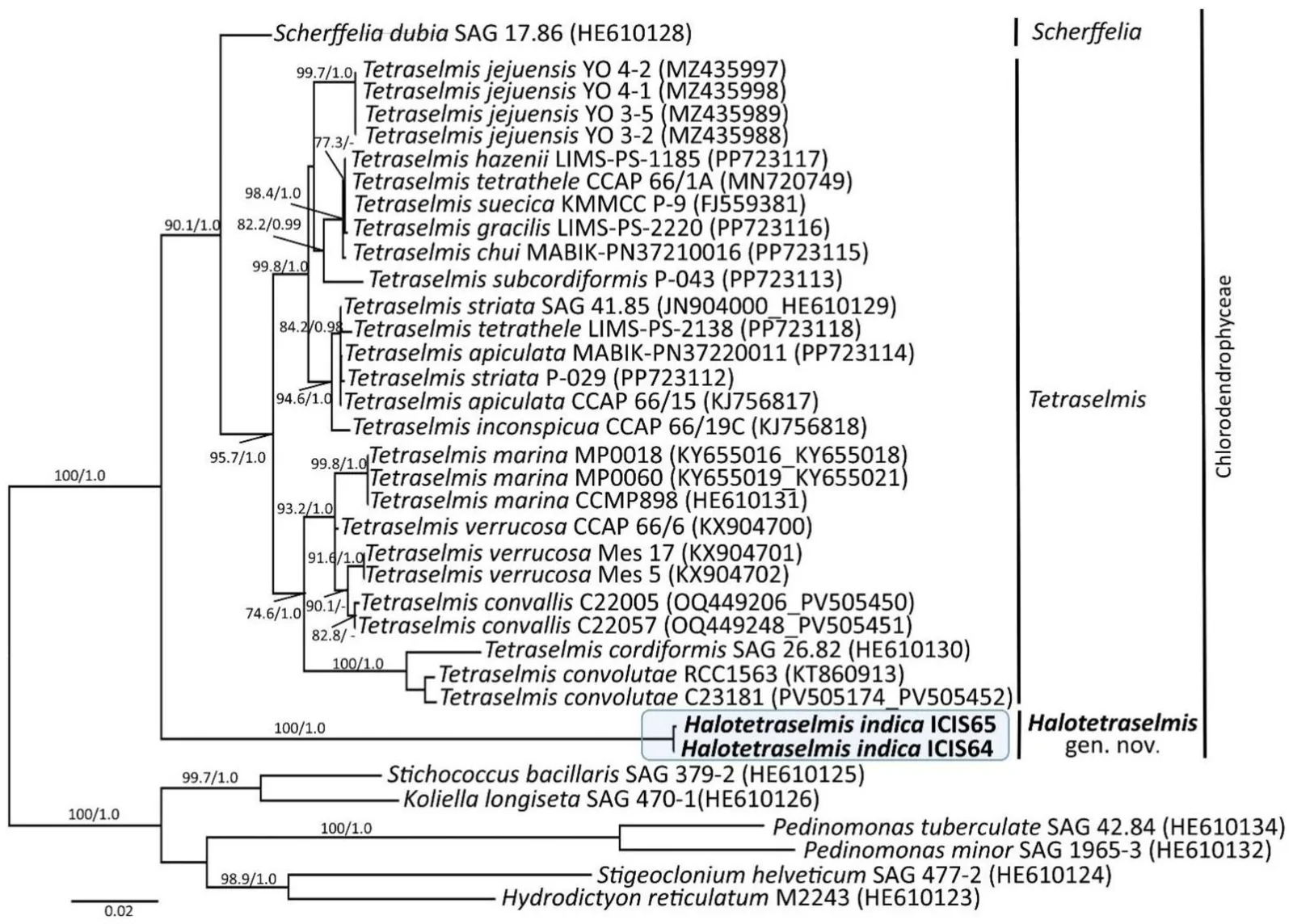
Maximum-likelihood tree of Chlorodendrophyceae inferred from concatenated 18S rRNA and 28S rRNA genes. Bootstrap values (BS) ≤ 70% and Bayesian Posterior Probability (BPP) values < 0.9 were omitted. The nucleotide sequences of *Pedinomonas minor SAG 1965-3*, *Pedinomonas tuberculate* SAG 42.84, *Hydrodictyon reticulatum* M2243, *Stigeoclonium helveticum* SAG 477-2, *Koliella longiseta* SAG 470-1, and *Stichococcus bacillaris* SAG 379-2 were used as the outgroup. Sequences generated in this study are in bold.

Phylogenetic analysis based on 28S rDNA sequences placed the new strains in a well-supported, distinct lineage within the class Chlorodendrophyceae, external to both *Tetraselmis* and *Scherffelia* (Figure S3). This basal position of the *Halotetraselmis* clade was further corroborated by analysis of the concatenated 18S–28S rRNA dataset, in which the *Halotetraselmis* lineage branched external to *Scherffelia* with the maximal statistical support (Figure 5).

Both the single-gene 28S rRNA phylogeny and the concatenated 18S–28S rRNA phylogeny recovered well-supported clades corresponding to *Tetraselmis* and *Scherffelia*, as well as most subclades within *Tetraselmis*, a resolution unattainable using the 18S rRNA gene alone. These results demonstrate that the 28S rRNA gene as a more informative phylogenetic marker for Chlorodendrophyceae than 18S rRNA. Notably, the intrageneric topology recovered for *Tetraselmis* in the concatenated 18S–28S rRNA tree is consistent with that obtained by the maximum-likelihood analysis of the concatenated 18S–ITS–28S dataset reported by Riewluang and Wakeman [27].

#### 3.2.3. *rbcL* Gene Phylogeny

**Figure 6:**
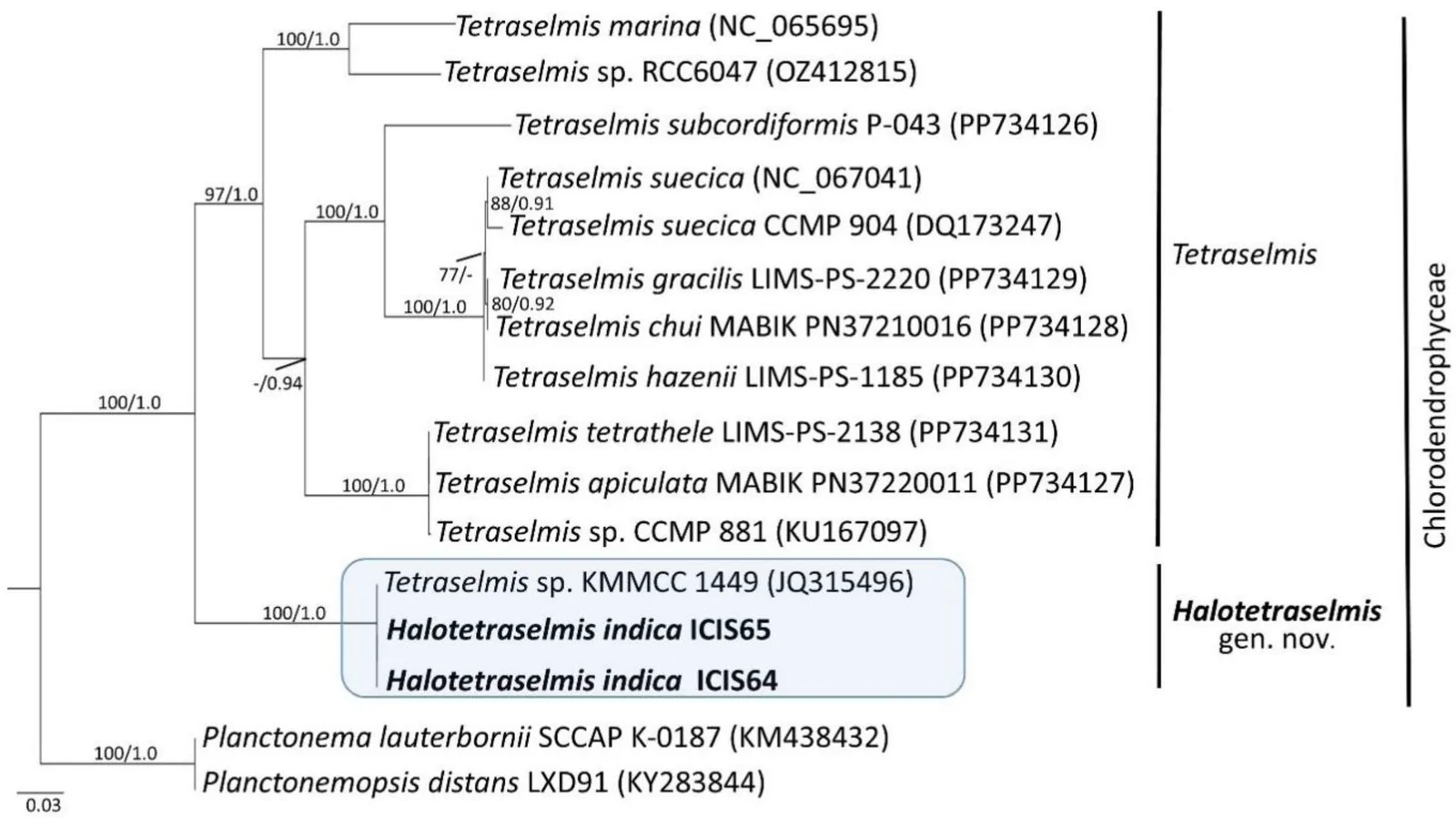
Maximum likelihood tree of Chlorodendrophyceae showing the phylogenetic position of the new strains ICIS64 and ICIS65, constructed based on the comparison of *rbcL* gene nucleotide sequences. The GTR + F + I model was selected as the optimal evolutionary model. Statistical support for tree nodes is shown as bootstrap and Bayesian posterior probability support values (BP/BPP); BP values ≤ 70% and BPP ≤ 0.9 are not shown. The nucleotide sequences of *Planctonema lauterbornii* and *Planctonemopsis distans* were used as the outgroup. The strains studied are shown in bold.

The gene encoding the large subunit of ribulose-1,5-bisphosphate carboxylase/oxygenase (*rbcL* gene) was partially sequenced for both strains ICIS64 and ICIS65 (1265 and 1216 bp, respectively). This genetic marker was sequenced for *Halotetraselmis indica* for the first time. The nucleotide sequences of the two strains were similar to each other and to the JQ315496 sequence from a culture isolated from seawater (Korea) and designated as *Tetraselmis* sp. KMMCC 1449. A phylogenetic analysis based on a comparison of all *rbcL* gene sequences of representatives of the class Chlorodendrophyceae available in GenBank showed with high statistical support that the *Halotetraselmis* clade, comprising strains ICIS64 and ICIS65, as well as *Tetraselmis* sp. KMMCC 1449, occupies an external position relative to the genus *Tetraselmis* (Figure 6). Notably, unlike the 18S rRNA gene, phylogenetic analysis based on the *rbcL* gene did not reveal differences between the ICIS strains and their closest homologs, placing them in a single clade. This marker therefore appears unsuitable for species-level discrimination, although it shows good resolution at higher taxonomic levels; in particular, the division of the genus into clades corresponding to the subgenera is consistent with their topology in the concatenated tree, and also reveals the polyphyly of *Parviselmis*. This contrast further supports the utility of *rbcL* for delimiting genus- and subgenus-level clades within Chlorodendrophyceae, while underscoring the need for additional markers, such as ITS2, to resolve species boundaries within *Halotetraselmis*.

#### 3.2.4. ITS1-5.8S-ITS2 Fragment Analysis

Given the high species-level resolving power of the ITS1-5.8S-ITS2 fragment as a genetic marker in green microalgae in general [69], as well as the importance of the ITS1 and ITS2 regions for distinguishing between cultures and species within the *Tetraselmis* genus [29], the ITS1-5.8S-ITS2 fragment was sequenced to further resolve the phylogenetic position of the new strains ICIS64 and ICIS65 (GenBank accession numbers PZ485853 and PZ485854, respectively). The sequences obtained for the two strains were completely identical. Phylogenetic analysis of the new strains together with representatives of *Tetraselmis* and *Scherffelia* based on these sequences could not be performed, as a BLAST search against the GenBank nucleotide database retrieved only four relatively closely related sequences with sufficient coverage (Table 2). Two of these belonged to cultured strains identified as *Tetraselmis* sp., while the other two were environmental sequences obtained from a hypersaline microbial mat in Puerto Rico that had been erroneously assigned to fungi [70]. Notably, the ITS2 sequence similarity between the studied strains and *Tetraselmis* sp. KMMCC 1449 (JQ315811) was only 82.26%, which, according to the species delimitation criterion proposed by Hoshina [71], whereby ITS2 divergence above 10% indicates separate species, is consistent with the studied strains and this reference isolate belonging to different species, despite the high similarity observed at the 18S rRNA and *rbcL* loci. Similarity with *Tetraselmis* sp. A5 (KU682841) was higher, at 95.56%; at this level of divergence (4.44%), which falls within Hoshina’s intermediate, non-diagnostic zone (2–10%), it is not possible to determine whether the two taxa represent the same or different species.

**Figure 7:**
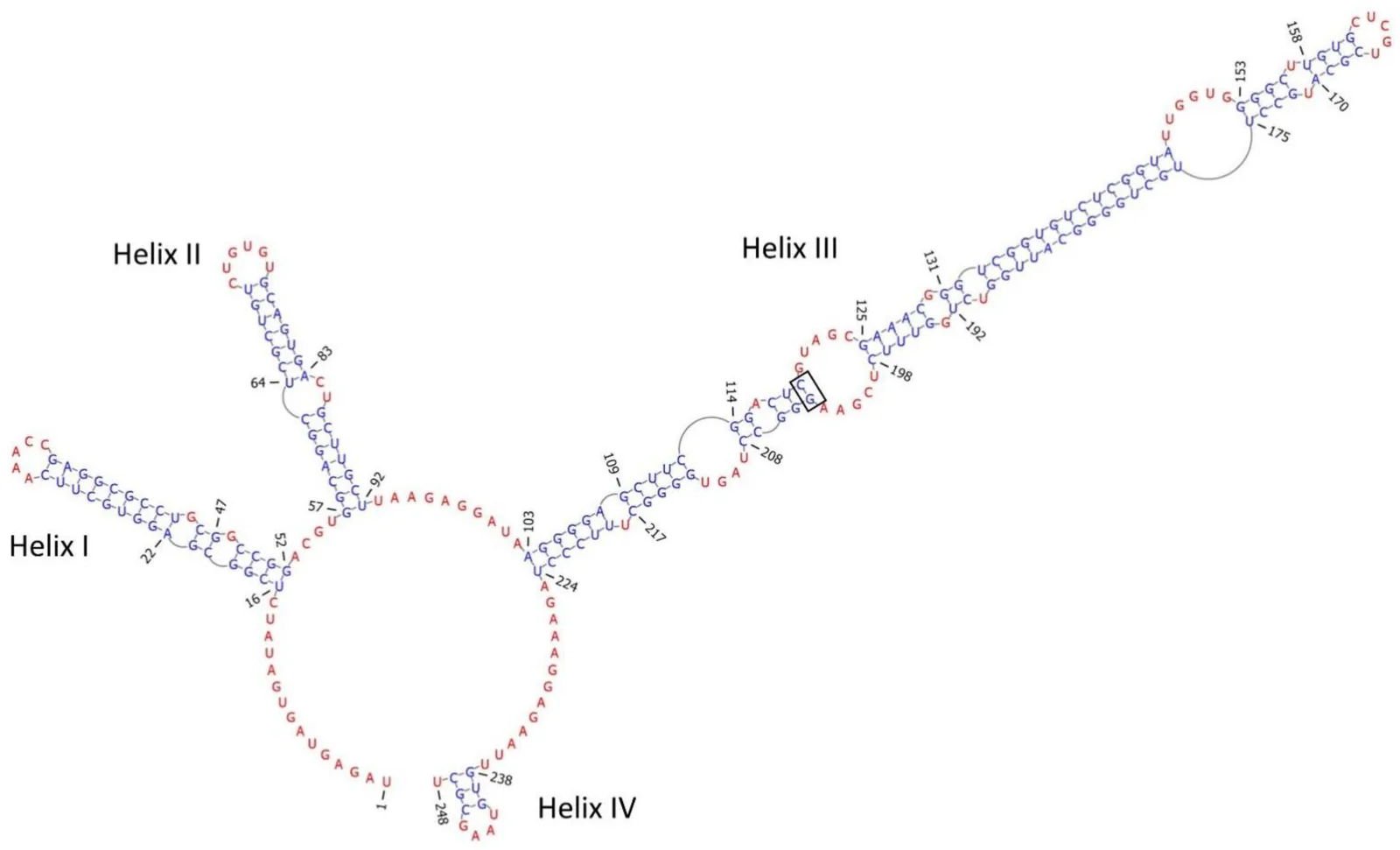
ITS2 secondary structure of *Halotetraselmis indica* (both ICIS64 and ICIS65 strains). CBC with “*Tetraselmis*” sp. A5 KU682841 is highlighted with a black rectangle.

Since the ITS2/CBC approach has been successfully applied for species delimitation in green microalgae, we reconstructed the secondary structure of the ITS2 region of the newly obtained sequences from the new strains and compared it with that of their closest homologs [72]. The ITS2 sequences of both ICIS strains were 248 nucleotides in length and folded into the typical cloverleaf secondary structure comprising four main helices, with helix III being the longest, as is characteristic of green algae (Figure 7). The characteristic UGGU motif was present at the apex of helix III; however, the pyrimidine–pyrimidine mismatch typically found in helix II was absent. This mismatch was likewise absent in the ITS2 sequences of the two closest homologs available in GenBank: KU682841 (deposited as *Tetraselmis* sp. A5, isolated from Red Sea water) and JQ315811 (deposited as *Tetraselmis* sp. KMMCC 1449, isolated from seawater in Korea). This indicates that the absence of the mismatch is a conserved structural feature of the genus *Halotetraselmis* rather than a strain-specific peculiarity (Figure S4).

The ITS2 sequence of *Tetraselmis* sp. A5 was 247 nucleotides in length, and its secondary structure differed from that of the ICIS strains by a single CBC in helix III (U–A→C–G at position 16), which, according to Coleman’s criterion, is indicative of separate species status (Figure 7 and Figure S4). The ITS2 sequence of *Tetraselmis* sp. KMMCC 1449 was 263 nucleotides long, 15–16 nucleotides longer than that of ICIS and *Tetraselmis* sp. A5 strains. Although no full CBC was detected between the new strains and *Tetraselmis* sp. KMMCC 1449, substantial structural differences in helix III were observed, including a greater length and an earlier onset of the helix, a CBC-like substitution and numerous hemi-CBCs (hCBCs) [73] across all four helices (Figure S4). However, the absence of a full CBC does not rule out species-level divergence between the compared taxa, since Coleman’s criterion is asymmetric: the presence of a CBC provides strong support for distinct species status, whereas its absence does not necessarily indicate conspecificity.

### 3.3. Halotolerance of ICIS64 and ICIS65 Strains

**Figure 8:**
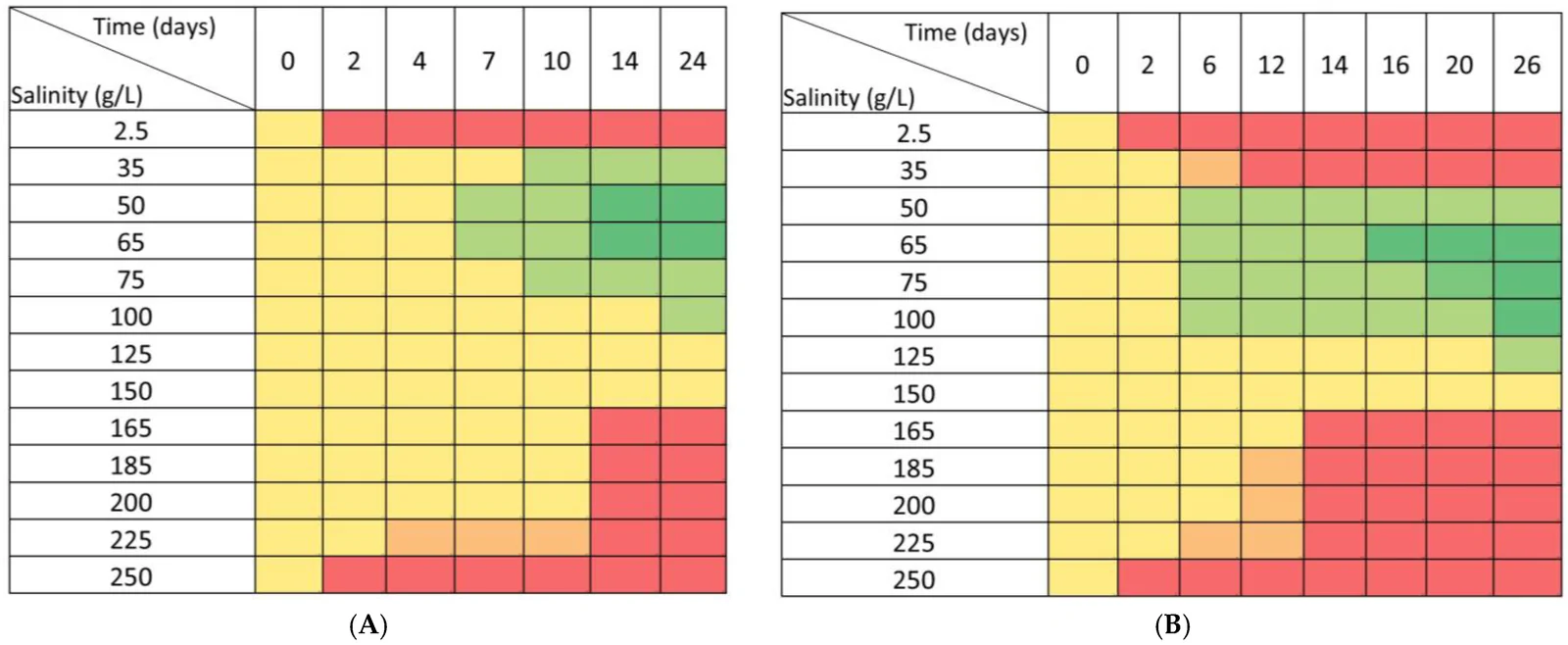
Heatmap illustrating the halotolerance limits and optimal salinity range for growth of *Halotetraselmis indica* strains ICIS64 (**A**) and ICIS65 (**B**). Color coding corresponds to the semi-quantitative scoring scale described in Materials and Methods: red—complete culture death (no living cells present in the entire well); orange—partial culture death (presence of solitary, non-flagellated cells when the entire well was examined); yellow—score 1 (1–10 cells in 10 fields of view); light green—score 2 (≥10 and <100 cells in 10 fields of view); dark green—score 3 (≥100 cells in 10 fields of view).

**Figure 9:**
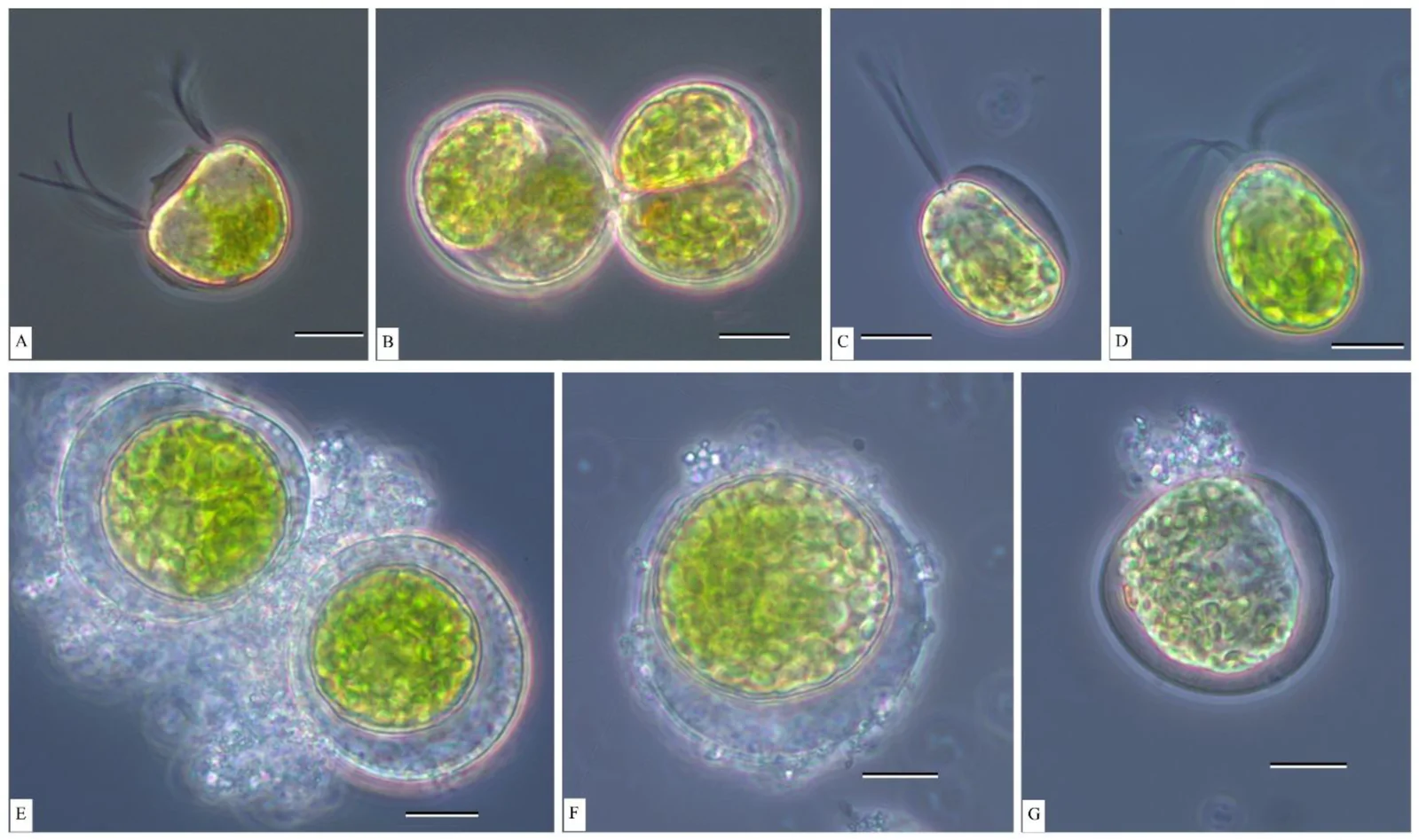
Light micrographs of *Halotetraselmis indica* ICIS64 cells cultivated at different salinities: 50 g/L (**A**), 100 g/L (**B**,**C**), 125 g/L (**D**,**E**), 150 g/L (**F**), 165 g/L (**G**) on day 10 of cultivation. (**A**) division of a motile cell; (**B**) division of non-motile cells; (**C**,**D**) vegetative cells; (**E**) resting cell; (**F**) resting cell with a thickened cell wall; (**G**) a dying cell. Scale bar = 10 μm.

Halotolerance analysis revealed that both strains are obligate halophiles. Exposure to a low salinity of 2.5 g/L resulted in a complete loss of vegetative cell viability within 48 h, with no cysts formation under these conditions (Figure 8). Both strains remained viable for extended periods at a salinity of 150 g/L, whereas at higher salinities (165–225 g/L) cultures died completely by day 14 of observation; during this decline, non-motile cell forms gradually lost their green coloration, first turning pale yellow and subsequently becoming colorless (Figure 9G). A salinity of 250 g/L or higher caused immediate death in both cultures.

The lower limit of halotolerance, however, differed between strains: *H. indica* ICIS64, isolated from a solar saltern near Sivash Bay, showed weak growth already at seawater salinity (35 g/L) (Figure 8A), whereas *H. indica* ICIS65, isolated from the hypersaline Malaya Smorogda River, began to grow at a salinity of 50 g/L (Figure 8B). Accordingly, cell proliferation was recorded within a salinity range of 35–100 g/L for strain ICIS64 (Figure 9A,B), with an optimum of 50–65 g/L (Figure 8A), while for strain ICIS65 the proliferation range was 50–125 g/L, with an optimum salinity of 65–75 g/L (Figure 8B).

At 150 g/L, both cultures persisted solely as non-motile, resting cells with thickened cell walls, densely colonized by satellite bacteria (Figure 9F), with motile cells entirely absent. At salinities of 125 g/L and below, both motile and non-motile cell forms were present (Figure 9C–E). These findings indicate strain-specific differences in the range of halotolerance.

### 3.4. Distribution of Halotetraselmis indica in Water Bodies Across Different Geographic Locations Based on ITS2 Metabarcoding Data

Among all amplicon sequence variants (ASVs) obtained from ITS2 metabarcoding data of hypersaline water bodies, four unidentified ASVs showed the highest similarity (99.7–100.0%) to the ITS2 sequences determined for strains ICIS64 and ICIS65. The occurrence and relative abundance of these four amplicon variants were assessed across 60 samples from 30 hypersaline water bodies at four geographic localities (Crimean Peninsula, Kulunda Steppe, and the Volgograd and Orenburg regions of Russia), with salinities ranging from 60 to 380 g/L and pH 6.8–10.7. The ASVs in question were detected in 7 of the 60 hypersaline samples examined (occurrence frequency of 11.7%), corresponding to 4 of the 30 hypersaline water bodies studied, located within two of the four geographic localities examined (Crimean Peninsula and Volgograd Region). The highest mean relative abundance of ASVs assigned to *Halotetraselmis indica*, reaching up to 26.44% of all microalgal ASVs in individual samples, was recorded at the mouth of the river Malaya Smorogda (salinity 90 g/L), which flows into Lake Elton (Table 3). A comparatively high relative abundance (up to 16.77%) was also recorded in Lake Yarylgach, with a salinity of 80 g/L, located in Crimea (Table 3). In the remaining samples, the target sequences were present at markedly lower abundances: ≤0.38% in an ephemeral pool near Lake Tobechikskoe (Crimea; salinity 360 g/L) and 0.01% in an ephemeral pool near Chokrak Lake (salinity 158 g/L) (Table 3). ASVs of *Halotetraselmis indica* were absent from the Sol-Iletsk salt lakes of the Orenburg Region and the soda lakes of the Kulunda Steppe. Overall, the presence of these ASVs was detected across a salinity range of 80–360 g/L and a pH range of 7.0–8.3.

ITS2 metabarcoding data on the occurrence and relative abundance of ASVs assigned to *Halotetraselmis* were compared with previously obtained 18S metabarcoding data, some of which were published [45,46]. Although 18S is a more conservative, less species-specific marker for detecting the presence of the taxon, these data confirmed the presence of ASVs assigned to *Halotetraselmis* in the same samples: the Malaya Smorogda River, Lake Yarylgach, and ephemeral pools near Lake Tobechikskoe and Chokrak Lake, as well as their predominance among all Chlorophyta in samples from the Malaya Smorogda River and Lake Yarylgach. However, the 18S dataset was considerably broader, comprising 136 samples from 47 water bodies with salinities ranging from 0 to 380 g/L and pH from 6.8 to 10.7. This allowed us to further identify the presence of ASVs assigned to *Halotetraselmis* in water bodies with salinities below 60 g/L, among which intermittent occurrence at low relative abundances was recorded only in two rivers flowing into Lake Elton, the Solyanka and Bolshaya Smorogda rivers (Table 3). Accordingly, in this expanded dataset, ASVs assigned to *Halotetraselmis* were detected in 12 of the 136 samples examined (8.82%), belonging to 6 of the 47 water bodies studied. Furthermore, these data demonstrate the potential for near-dominance of *H. indica* among chlorophytes; its relative abundance exceeded 90% in a 2014 sample from the Malaya Smorogda River at a salinity of 85 g/L [45]. Taken together, these metabarcoding data indicate that this taxon is rare in the studied dataset. Although its DNA was detected across a salinity range of 19–360 g/L at near-neutral pH, high relative abundance was recorded only in a narrow salinity range of 80–90 g/L, indicating pronounced ecological specificity attributable to selective association with particular environmental conditions.

### 3.5. Taxonomic Treatment

*Halotetraselmis* Selivanova, Tynnikov & Temraleeva, gen. nov.

Diagnosis. Differs from *Tetraselmis* in its distinct phylogenetic position, as well as in overall cell morphology, location of eyespot below pyrenoid level, rectangular shape of nucleus, abundant hairs of two types in apical aperture, pigment composition (absence of loroxanthin), and its hypersaline habitat. Differs from *Scherffelia* in its distinct phylogenetic position, overall cell morphology, the presence of a single cup-shaped chloroplast with a pyrenoid, and its hypersaline habitat.

Description. Halophilic planktonic green microalga, preferring salinities of 50–100 g/L (minimum salinity for growth: 35 g/L). Motile cells slightly compressed, bilaterally symmetrical, oval to broadly ovoid in outline when viewed from the broad side, elliptical when viewed from the narrow side, with an invagination at the anterior end and four equal flagella arranged in two opposing pairs. An eyespot (stigma) is present in the posterior third of the cell. The cell is enclosed by a theca composed of a layer of aggregated organic scales. The nucleus, located in the anterior half of the cell, is rectangular to shield-shaped, with an apical groove and a basal arch. In addition to the motile stage, the life cycle also includes a non-motile stage and resting cells with thickened cell walls. Reproduction occurs by division within the parental theca, producing two or four daughter cells; daughter cells develop flagella while still surrounded by the parental theca. Cells occasionally divide asymmetrically.

Type species. *Halotetraselmis indica* (M.Arora & A.Anil) Selivanova, Tynnikov & Temraleeva, comb. nov.

Etymology. *Halo-* [Greek, ἅλς, salt] + *Tetraselmis*, in reference to the obligately halophilic nature of the type species and its close phylogenetic relationship to the genus *Tetraselmis*, within which it was originally described.

*Halotetraselmis indica* (M.Arora & A.Anil) Selivanova, Tynnikov & Temraleeva, comb. nov.

Basionym. *Tetraselmis indica* M.Arora, A.Anil, F.Leliaert, J.Delany & E.Mesbahi [18].

Holotype. Permanently resin-embedded strain deposited in the National Facility for Marine Cyanobacteria, Bharathidasan University, Tiruchirappalli, Tamil Nadu, 620024, India (Accession Number BDU GD001), designated as *Tetraselmis indica* M.Arora, A.Anil, F.Leliaert, J.Delany & E.Mesbahi in the protologue [18]; GenBank accession No. HQ651184 for the 18S rRNA gene sequence. Type locality: salt pan near Mandovi Bridge, Panaji, Goa, India (15.500623° N, 73.849046° E).

Epitype (designated here). ICIS64, deposited as VKM Al-571 in the All-Russian Collection of Microorganisms. The holotype is demonstrably ambiguous for the precise application of the name, as its molecular characterization is limited to the 18S rRNA gene, which Arora et al. (2013) [18] themselves noted was insufficient to precisely resolve the phylogenetic placement of the species within the genus. The epitype is designated here to supplement the holotype with a multilocus molecular dataset (18S rRNA gene, ITS1-5.8S-ITS2 region, 28S rRNA gene, and *rbcL* gene) necessary for the precise and stable application of the name *Halotetraselmis indica*. Epitype locality: saltern near Sivash Bay, Crimea (45°19′43″ N, 35°24′01″ E).

Diagnosis. Differs from species of *Tetraselmis* in 18S rRNA gene and ITS1-5.8S-ITS2 sequences, as well as in ITS2 secondary structure.

Description (emended). Description below is emended from the original protologue [22], based on re-examination of epitype material and newly acquired morphological, physiological, and molecular data. Halophilic planktonic green alga, preferring high salinities (50–100 g/L) and remaining viable for extended periods at salinities from 35 to 150 g/L. Motile cells slightly compressed, 17.4–28.7 µm long (mean 22.1 ± 0.2 µm), 15.6–25.0 µm wide (mean 19.2 ± 0.2 µm), and 10.9–18.2 µm thick (mean 14.8 ± 0.3 µm) under optimal salinity conditions (70 g/L). Cells are oval to broadly ovoid in outline when viewed from the broad side, elliptical when viewed from the narrow side, with an apical invagination (a furrow passing from one broad face to the other) and four equal flagella arranged in two opposing pairs. Flagella are covered along their entire length by numerous fine hairs approximately 1.0–1.5 µm long and scales arranged in regular rows, closely abutting one another diagonally. Cells contain a single cup-shaped chloroplast, ranging in color from dark green to yellowish-green, with a large, conspicuous pyrenoid; the pyrenoid is located in the posterior third of the cell, its matrix traversed from several directions by cytoplasmic canaliculi, 2.6–2.8 × 3.4–3.6 µm in longitudinal section, surrounded by numerous starch grains, mostly biconvex and occasionally concave on one side. A red-orange stigma (one, or occasionally several) is located below the level of the pyrenoid, in the posterior third of the cell. In addition to the motile stage, the life cycle also includes a non-motile stage and resting cells with thickened cell walls, allowing temporary survival at salinities above 150 g/L. Asexual reproduction occurs by division within the parental theca, producing two or four daughter cells; daughter cells develop flagella while still surrounded by the parental theca. Dictyosomes usually 2–8, positioned in a circle near the anterior end of the nucleus and sometimes laterally. Nuclear morphology, dimensions (shield-shaped, 6.5–8.5 µm long, 3.5–4 µm broad, with a characteristic apical groove and a basal arch, both visible in many longitudinal sections; nucleolus very prominent), and the distribution of the endoplasmic reticulum were consistent with the original description and were not re-examined in this study [18]. Loroxanthin, a pigment otherwise characteristic of the genus *Tetraselmis*, is absent from the pigment complement of *H. indica* [41].

Habitat. Hypersaline conditions, with a halotolerance range of 35–150 g/L and optimal growth salinity of 50–100 g/L, persist at salinities above 150 g/L through the formation of resting cells.

Material examined. Strain ICIS64 (VKM Al-571), isolated from brine of the Sivash Bay saltern, Crimea (45°19′43″ N, 35°24′01″ E), sampled in August 2021: PZ477976 (18S rRNA gene), PZ639060 (28S rRNA gene), PZ485853 (ITS1-5.8S-ITS2), and PZ495605 (*rbcL*). Strain ICIS65 (VKM Al-570), isolated from brine of the hypersaline Malaya Smorogda River, Volgograd Region, Russia (49°05′46″ N, 46°43′59″ E), which flows into Lake Elton, sampled in August 2018: PZ477977 (18S rRNA gene), PZ639059 (28S rRNA gene), PZ485854 (ITS1-5.8S-ITS2), and PZ495606 (*rbcL*).

Remarks. *Tetraselmis indica* was originally described within the genus *Tetraselmis* [18], but its phylogenetic placement could not be resolved on the basis of 18S rRNA gene data alone, and the species showed a large genetic distance from all other described members of the genus. Multilocus molecular phylogenetic analysis (18S rRNA gene, ITS1-5.8S-ITS2 region, 28S rRNA gene, and *rbcL* gene) of newly isolated strains closely related to *T. indica* (ICIS64 and ICIS65) confirms and substantially strengthens this earlier observation, resolving a well-supported clade external to *Tetraselmis*. This result is corroborated by chemotaxonomic evidence, specifically the absence of loroxanthin, a pigment otherwise characteristic of *Tetraselmis* [41]. We therefore consider the genetic, morphological, and chemotaxonomic distinctness of this lineage sufficient to warrant recognition as a separate genus, *Halotetraselmis* gen. nov.

## 4. Discussion

### 4.1. Genus-Level Distinctness of Halotetraselmis

Delimitation of *Halotetraselmis* as a new genus, rather than a new species, is supported by a polyphasic study of two newly isolated halophilic strains (ICIS64 and ICIS65) from geographically distant hypersaline habitats in Crimea and the Lake Elton basin. Multilocus analysis resolved the taxon’s phylogenetic position, previously left unresolved [18]. *Halotetraselmis* was consistently recovered as an independent, well-supported, early-diverging lineage, rather than a subclade nested within *Tetraselmis* or *Scherffelia*, across both single- and multi-gene phylogenies, providing strong molecular support for generic status. This is consistent with genus-level delimitation criteria applied within Chlorophyta [74,75,76,77], and is reinforced by genetic distances between *Halotetraselmis* and two other Chlorodendrophyceae genera exceeding the established inter-generic benchmark by two- to three-fold (this study). These results are consistent with previously identified differences in cell ultrastructure [18] and pigment composition (absence of loroxanthin), as well as with its hypersaline habitat, uncharacteristic of other Chlorodendrophyceae [41]. Together, these features form a diagnostic character combination that reliably distinguishes *Halotetraselmis* from other genera. Such large genetic distances could suggest a higher taxonomic rank, at least at the level of family. However, given the incomplete taxonomic revision of Chlorodendrophyceae and limited data on *Halotetraselmis* [27], we consider this premature.

The ITS1-5.8S-ITS2 region could not be included in the concatenated dataset, as was done by Riewluang & Wakeman [27], owing to its pronounced divergence between *Halotetraselmis* and *Tetraselmis*, further supporting their phylogenetic distance. Nevertheless, ITS2 similarity combined with the CBC approach, widely used for species delimitation in Chlorophyta [71], clarified relationships between our strains and related, unidentified strains within the *Halotetraselmis* clade. This suggests that the sister clade, comprising *Tetraselmis* sp. GSL018 (Great Salt Lake, USA [18]) and *Tetraselmis* sp. KMMCC1449 (Korea), may represent a species distinct from *H. indica*. A CBC in helix III of the ITS2 secondary structure of an undescribed Red Sea strain, relative to our strains, likewise indicates distinct species status. Thus, *Halotetraselmis* appears to comprise at least three species, a hypothesis requiring confirmation based on the polyphasic characterization of these and other strains. The ITS1-5.8S-ITS2 sequences and ITS2 secondary structure obtained in this study serve as a starting point for intrageneric differentiation.

### 4.2. Different Genetic Marker Resolution

Differences in the resolution of genetic markers reflect significant variations in their evolutionary rates, as previously noted [78]. Relying solely on the 18S rRNA gene proved insufficient to resolve the branching position of *Halotetraselmis* and many other lineages within the class Chlorodendrophyceae, although the separation into two distinct clades corresponding to separate species was clearly evident. The 28S rRNA gene, whether used alone or in combination with the 18S gene, strengthened the phylogeny and provided robust resolution for most branches; the resulting topology for the *Tetraselmis*/*Scherffelia* group matched that obtained from analysis of the complete ribosomal operon [27]. In contrast, intergenic spacer regions showed extreme divergence between *Halotetraselmis* and *Tetraselmis* and proved informative only for species delimitation among close relatives. The *rbcL* gene allowed for reliable resolution of topology at the genus and subgenus levels but failed to resolve species-level relationships within the genus *Halotetraselmis*, indicating a higher degree of conservation compared to nuclear markers [78].

This pattern is consistent with slower evolution of core metabolic genes, such as those for photosynthesis, in organisms with behavioral flexibility for coping with stress. One possible explanation for the low divergence of *rbcL* among *Tetraselmis* species is a hypothesis that links microalgal motility to the evolution of the photosynthetic apparatus [79]: phototaxis may reduce selective pressure on the Rubisco activase system, as the organism’s ability to actively select favorable conditions renders the current enzyme functionally sufficient, potentially resulting in a slower rate of molecular evolution of *rbcL*. Consequently, *rbcL* is used less often as a standalone marker in this group, appearing more frequently in broader chloroplast phylogenomic datasets for core Chlorophyta [12,30] than in species-level studies of *Tetraselmis*.

The consistent recovery of *Halotetraselmis* as a well-supported clade external to *Tetraselmis*, across markers of markedly different evolutionary rates, from the conserved 18S rRNA and *rbcL* genes to the more variable 28S rRNA gene, indicates that this result reflects genuine, long-standing evolutionary separation rather than a single-locus artifact (Figure 5, Figure 6 and Figure S3). *Halotetraselmis* appears to have diverged before the split of *Tetraselmis* and *Scherffelia*, consistent with an evolutionary trajectory sufficiently deep to warrant generic distinction. This places the origin of obligate halophily in this lineage at a phylogenetically earlier-diverging position than previously assumed [18].

### 4.3. Species-Level Distinctness of Halotetraselmis indica

We found no justification for describing a new species based on the newly isolated ICIS strains, as they were identical to the “*Tetraselmis indica*” strain MA-2011 in 18S rRNA gene sequence and had similar morpho-physiological characteristics. The only morphological difference was a larger minimum cell size in our strains, though the size ranges overlapped. This may reflect the marine salinity used to cultivate MA-2011 [18], suboptimal according to our data compared with the higher salinity under which our strains were maintained. This suboptimal, physiologically stressful salinity is consistent with the conditions under which Arora et al. [40] reported asymmetric division, whereas in our strains such division was observed only rarely, at 70 g/L. We therefore attribute the smaller minimum cell size reported by Arora et al. [18] to increased population heterogeneity from asymmetric division under stress, rather than to a taxonomically relevant difference between strains.

We also supplemented the description with additional details. Scanning electron microscopy revealed a characteristic rhomboidal (diamond-shaped) arrangement of flagellar scales, previously described only by TEM [18]. We further added morphometric data on resting cells, noting a wide gap between the theca and protoplast, thicker cell walls than in vegetative cells, and possible multiple theca layers, the latter consistent with subsequent findings by the same group [35]. We also provide physiological data, namely the halotolerance limits and salinity optimum of vegetative cells, both determined experimentally, which partly account for the species’ detection pattern in natural samples.

### 4.4. Ecological Characteristics and Geographical Distribution

According to its halotolerance range, *H. indica* is a truly obligate halophile, joining a rather limited group of green algae capable of sustained active growth in hypersaline habitats [80]. Obligate halophilic representatives are known within the class Chlorophyceae, namely the genus *Dunaliella*, including *D. salina*, *D. viridis*, and *D. parva*, found worldwide in salt lakes and in saltern evaporation and crystallizer ponds at salt concentrations up to NaCl saturation [81], and members of the genus *Asteromonas*, namely *Asteromonas gracilis*, described from salt lakes and salt marshes at salinities ranging from 29.25 to 321.75 g/L. Among the tiny algae of Picocystophyceae, *Picocystis salinarum* is known to be characteristic of soda lakes [82]. Among Trebouxiophyceae, some species, such as *Picochlorum renovo*, exhibit broad halotolerance, ranging from 8.75 to 107.5 g/L [83]. Hypersaline water bodies are extreme ecosystems characterized by low species diversity, in which halophilic microalgae serve as the main primary producers, driving the microbial loop and supporting heterotrophic communities [84]. While the genus *Dunaliella* dominates at extremely high salinity levels exceeding 200 g/L [46,81], *Halotetraselmis* occupies a distinct and narrow ecological niche under conditions of moderate hypersalinity (80–100 g/L). The ability of this organism to proliferate in such transitional zones and to form mass blooms there highlights its significant contribution to carbon fixation and organic matter production in unstable thalassohaline and athalassohaline habitats.

Vegetative cells remain viable for only a short time at salinities exceeding 150 g/L and are incapable of forming thick-walled cysts. The thin-walled, non-motile cells that develop under such conditions resemble the resting cells formed during prolonged exposure to darkness [35]; these, too, die off by the 14th day. However, cysts formed at lower salinities are likely capable of withstanding significant subsequent fluctuations in salinity, a fact that may explain the detection of *H. indica* in environments with salinities up to 350 psu [18] and up to 360 g/L (based on metabarcoding data for the 18S and ITS2 regions; sample from a temporary water body near Lake Tobechikskoye). For instance, strain ICIS64 was isolated as resting cells from a sample with a salinity of 160 g/L. Notably, *H. indica* has been recorded specifically in hypersaline environments with unstable salinity: in salterns, in ephemeral shallow ponds where salinity rises due to evaporation and drops after heavy rains, and in rivers and lakes with fluctuating salinity [85]. Thus, the presence of resting cells enables the species to survive such instability but does not support continuous growth under conditions of constantly extreme salinity, where the species is absent. Despite its relative rarity (the species was detected at only two of the four localities examined and in far fewer water bodies otherwise suitable in terms of other parameters), *H. indica* is capable of massive blooms at 80–90 g/L. For instance, in the Malaya Smorogda River, at a salinity of 85 g/L, it accounted for over 90% of the algal community [45]. This pattern—combining rare occurrence, rapid blooming under favorable salinity, and persistence in extreme conditions via resting cells—reflects a combined stress-tolerant and ruderal (S-R) strategy [86], similar to that of *Asteromonas gracilis* [87]. However, given the scarcity of reference ITS2 sequences for halophilic Chlorodendrophyceae in public databases, conclusions regarding distribution based solely on metabarcoding data should be interpreted with caution as a preliminary map requiring further verification through targeted culture isolation.

Combining cultivation methods with ITS2 metabarcoding has expanded our understanding of the *H. indica* distribution range, which now includes, in addition to the previously known site in Panaji, Goa, India (salt pan near the Mandovi River bridge) [18], the Crimean Peninsula (Sivash Bay salterns, Yarylgach Lake, and ephemeral pools near Lakes Tobechikskoe and Chokrak) and the Volgograd Region of Russia (hypersaline and saline rivers in the Lake Elton basin). This demonstrates its presence, despite its rarity, in both thalassohaline and athalassohaline habitats. Apart from these locations, published records place the genus worldwide: North America (Great Salt Lake), Central America (a Puerto Rican hypersaline microbial mat), coastal Korea, the Red Sea, Yuncheng Salt Lake, China [85], the salt ponds and salterns of Curaçao [88], and Spain (Maspalomas Dunes Special Nature Reserve, Gran Canaria, Canary Islands). Moreover, halophilic Chlorodendrophyceae have also been recorded in Australia. As most of these records rest on 18S metabarcoding or unidentified cultures, the ITS2 references generated here remove a key barrier to their identification, particularly given our evidence that *Halotetraselmis* likely comprises at least two additional, currently undescribed species related to *H. indica* (see above). Re-examining these phylotypes and strains through ITS2-based species delimitation may show that the apparent cosmopolitan distribution of *H. indica* in fact reflects the combined ranges of several distinct, still poorly characterized species within the genus.

The live cultures deposited in this study as VKM Al-570 and VKM Al-571 provide a resource for future whole-genome and organellar phylogenomic analyses, which will be necessary to resolve the still-unsettled phylogenetic position of Chlorodendrophyceae relative to other early-diverging lineages of core Chlorophyta [13].

## 5. Conclusions

*Halotetraselmis* gen. nov. and *H. indica* comb. nov. (Chlorodendrophyceae, Chlorophyta) were described based on newly isolated strains obtained from brine of the Sivash Bay saltern, Crimean Peninsula (epitype), and from brine of the hypersaline Malaya Smorogda River, Volgograd Region, Russia, using an integrative approach combining morphology, physiology (halotolerance and salinity optimum), and multilocus molecular phylogenetics (18S rRNA gene, ITS1-5.8S-ITS2 region, 28S rRNA gene, and *rbcL* gene). The early-diverging (basal) position of *Halotetraselmis* within Chlorodendrophyceae, along with the substantial genetic distances separating it from *Tetraselmis*, and its obligate halophily, combined with the previously described morphological and chemotaxonomic differences, consistently support recognition of this lineage at the new genus level. The detection of *H. indica* in geographically distant localities of the temperate zone of Eurasia (Crimea, the Lake Elton basin) broadens current understanding of the geographic distribution of halophilic Chlorodendrophyceae. In addition to demonstrating the diagnostic value of the multilocus approach for differentiation at the genus and subgenus levels, this study also showed that ITS2 sequences can be used to delimit species within the genus *Halotetraselmis* and allowed the generation of ITS2 reference sequences for use in ITS2 metabarcoding. These findings broaden the current understanding of the diversity, phylogeny, and ecology of the class Chlorodendrophyceae and demonstrate that the diversity of its halophilic representatives has been substantially underestimated, highlighting the incompleteness and need for a comprehensive taxonomic revision of the class taking into account these lineages.

## Figures and Tables

**Table 1:** Primers and amplification conditions.

Locus	Primer	Sequence (5′–3′)	Amplification Conditions	Reference
18S rRNA	EukA	AACCTGGTTGATCCTGCCAGT	98 °C, 3 min; 95 °C (30 s), 57 °C (30 s), 72 °C (1 min), 35 cycles; 72 °C, 5 min	[49]
	FAD4 (EukB)	TGATCCTTCTGCAGGTTCACCTAC		
	18SintF *	GGTAATTCCAGCTCCAATAGCGTA		[50]
	18SintR *	GTTTCAGCCTTGCGACCATACT		
ITS1-5.8S-ITS2	ITS5	GGAAGTAAAAGTCGTAACAAGG	98 °C, 3 min; 95 °C (30 s), 53 °C (30 s), 72 °C (1 min), 35 cycles; 72 °C, 5 min	[51]
	ITS4	TCCTCCGCTTATTGATATGC		
*rbcL*	M28F	GGTGTTGGATTWAAAGCTGGTGT		[52]
	M1390Ra	CTTTCCAAAYTTCACAAGCAGCAGCAG		
	M633F *	CATGCGTTGGAGAGACCGT		
	M650R *	CGGTCTCTCCAACGCATGA		
28S rRNA	D1RF1	ACCCGCTGAATTTAAGCATA	95 °C, 3 min; 95 °C (30 s), 52 °C (30 s), 72 °C (1 min), 35 cycles; 72 °C, 5 min	[27]
	852R-70 *	CGAACGATTTGCACGTCAG		
	28-1483R	GCTACTACCACCAAGATCTGC		

* Internal sequencing primers.

**Table 2:** Sequence similarity of the ITS1-5.8S-ITS2 complex between strains *Halotetraselmis indica* ICIS64 and ICIS65 and their closest homologs from GenBank.

Closest Homologs (Accession Number)	Sampling Site	Genetic Marker					
		ITS1-5.8S-ITS2		ITS1		ITS2	
		Query Cover (%)	Similarity (%)	Query Cover (%)	Similarity (%)	Query Cover (%)	Similarity (%)
*Tetraselmis* sp. A5 (KU682841)	Red Sea water	96.00	92.56	n/d	n/d	100.00	95.56
*Tetraselmis* sp. KMMCC 1449 (JQ315811)	seawater (Korea)	100.00	86.30	99.0	84.00	99.00	82.26
Uncultured fungus clone PR-MAT-CV5-7 (FJ265947)	Puerto Rican subtropical hypersaline microbial mat	100.00	86.45	97.0	85.28	100.00	81.60
Uncultured fungus clone PR-MAT-CV5-6 (FJ265946)		99.00	86.05	97.0	85.28	99.00	80.57

n/d—no data.

**Table 3:** Characteristics of the hypersaline water bodies in which ASVs assigned to *Halotetraselmis indica* were detected, based on 18S and ITS2 metabarcoding data.

Water Body (Sampling Point)	Coordinates	Date	Salinity (g/L)	pH	Relative Abundance (%) of *Halotetraselmis indica* ASVs Among Total Chlorophyta ASVs	
					18S	ITS2
Rivers flowing into Lake Elton (Volgograd Region)						
Malaya Smorogda River (mouth)	N 49.095693, E 46.734580	25 August 2014	85	8.49	96.56–97.05 *	n/d
		18 August 2018	90	8.01	6.60–51.07 *	8.46–26.44
Solyanka (mouth)	N 49.178959, E 46.591594	18 August 2018	30	7.04	0–0.21 **	n/d
Bolshaya Smorogda River (mouth)	N 49.127463, E 46.787351	25 August 2014	19	7.91	0.20–0.55 *	n/d
		18 August 2018	14	7.48	0 **	n/d
Crimean Peninsula						
Yarylgach Lake	N 45.568464, E 32.855528	16 July 2019	80	8.26	12.46–13.85 *	5.63–16.77
Ephemeral pool near Tobechikskoe Lake	N 45.185076, E 36.301733	10 July 2019	360	7.00	0.07–1.02 **	0.38–0.37
Ephemeral pool near Chokrak Lake	N 45.461916, E 36.312935	12 July 2019	158	7.40	0.08–0.10 **	0.01

n/d—No data. *—According to published data [45,46]. **—According to unpublished data.

## Data Availability

The studied strain ICIS64, isolated from brine of the Sivash Bay saltern, Crimean Peninsula, is deposited in the All-Russian Collection of Microorganisms (VKM Al-571); the studied strain ICIS65, isolated from brine of the hypersaline Malaya Smorogda River, Volgograd Region, Russia, is also deposited in the All-Russian Collection of Microorganisms (VKM Al-570). All sequences obtained for the strain ICIS64 (VKM Al-571) are deposited in GenBank under accession numbers: PZ477976 (18S rRNA gene), PZ639060 (28S rRNA gene), PZ485853 (ITS1-5.8S-ITS2), and PZ495605 (*rbcL*). All sequences obtained for the strain ICIS65 (VKM Al-570) are deposited in GenBank under accession numbers: PZ477977 (18S rRNA gene), PZ639059 (28S rRNA gene), PZ485854 (ITS1-5.8S-ITS2), and PZ495606 (*rbcL*). Raw sequence data of ITS2 metabarcoding have been deposited in the NCBI Sequence Read Archive under BioProject accession PRJNA1475234 (Biosample accessions SAMN60634155–SAMN60634214).

## Supplementary Materials

The following supporting information can be downloaded at: https://www.mdpi.com/article/10.3390/microorganisms14102224/s1, Figure S1: Micrographs of strain ICIS65 cells obtained using scanning electron microscopy; Figure S2: Maximum likelihood tree of Chlorodendrophyceae showing the phylogenetic position of the new strains ICIS64 and ICIS65, constructed based on comparison of 18S rRNA gene nucleotide sequences, constructed with MEGA11; Figure S3: Maximum likelihood tree of Chlorodendrophyceae showing the phylogenetic position of the new strains ICIS64 and ICIS65, constructed based on comparison of 28S rRNA gene nucleotide sequences (1379 bp); Figure S4: Differences in ITS2 secondary structure between *Halotetraselmis indica* comb. nov. and their closest homologues. Table S1: List of nucleotide sequences used in phylogenetic analysis of Chlorodendrophyceae; Table S2: Estimates of evolutionary divergence between sequences of the 18S rRNA gene; Table S3: Estimates of evolutionary divergence between sequences of the 28S rRNA gene.

## Abbreviations

The following abbreviations are used in this manuscript:

DNA	Deoxyribonucleic acid
RNA	Ribonucleic acid
ITS	Internal Transcribed Spacer
SSU	Small Subunit
PCR	Polymerase Chain Reaction
BLAST	Basic Local Alignment Search Tool
SEM	Scanning Electron Microscopy
TEM	Transmission Electron Microscopy
CBC	Compensatory Base Change
ML	Maximum Likelihood
BIC	Bayesian Information Criterion
GTR	General Time Reversible (nucleotide substitution model)
BP	Bootstrap Probability
BPP	Bayesian Posterior Probability
UTC clade	Ulvophyceae–Trebouxiophyceae–Chlorophyceae clade
PSU	Practical Salinity Unit
