# Supplementary material for: Halotetraselmis gen. nov. and Halotetraselmis indica comb. nov. (Chlorodendrophyceae, Chlorophyta): New Halophilic Green Algae from Geographically Distant Hypersaline Habitats
Source: Microorganisms. 2026 Oct 2;14(10):2224. doi: 10.3390/microorganisms14102224 (PMC13707736; doi:10.3390/microorganisms14102224)
Supplement: Supplementary file 1 [file microorganisms-14-02224-s001.zip › Supplementary Materials_Figures S1-S4 and Table S1.pdf]

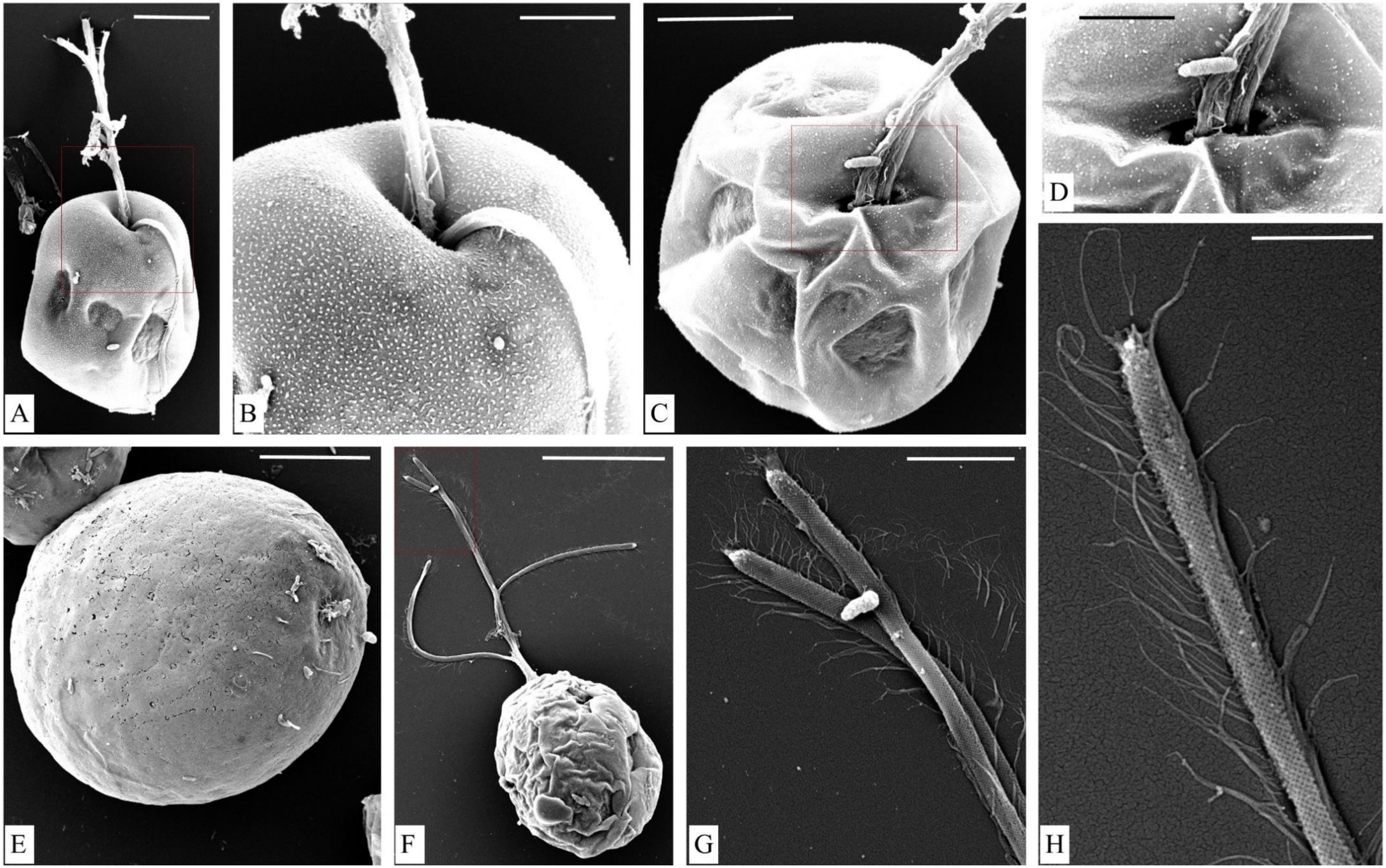

Figure S1. Micrographs of strain ICIS65 cells obtained using scanning electron microscopy. A – lateral view of flagellate cell covered externally by the theca; B – magnified micrographs showing the depression on the apical side; C – apical view of the emerging flagella; D – magnified micrographs showing a slit in the depth of the apical depression at the anterior part of the cell; E – resting cell with a dense wall and bacteria attached to its surface (the apical papilla, marking the former site of flagellar attachment, is visible); F – paired arrangement of the flagella and their general appearance; G, H – magnified micrographs of the flagella covered with scales and thin hairs. Scale bars: E, F — 10 µm; A, C — 5 µm; B, D, G — 2 µm; H — 1 µm.

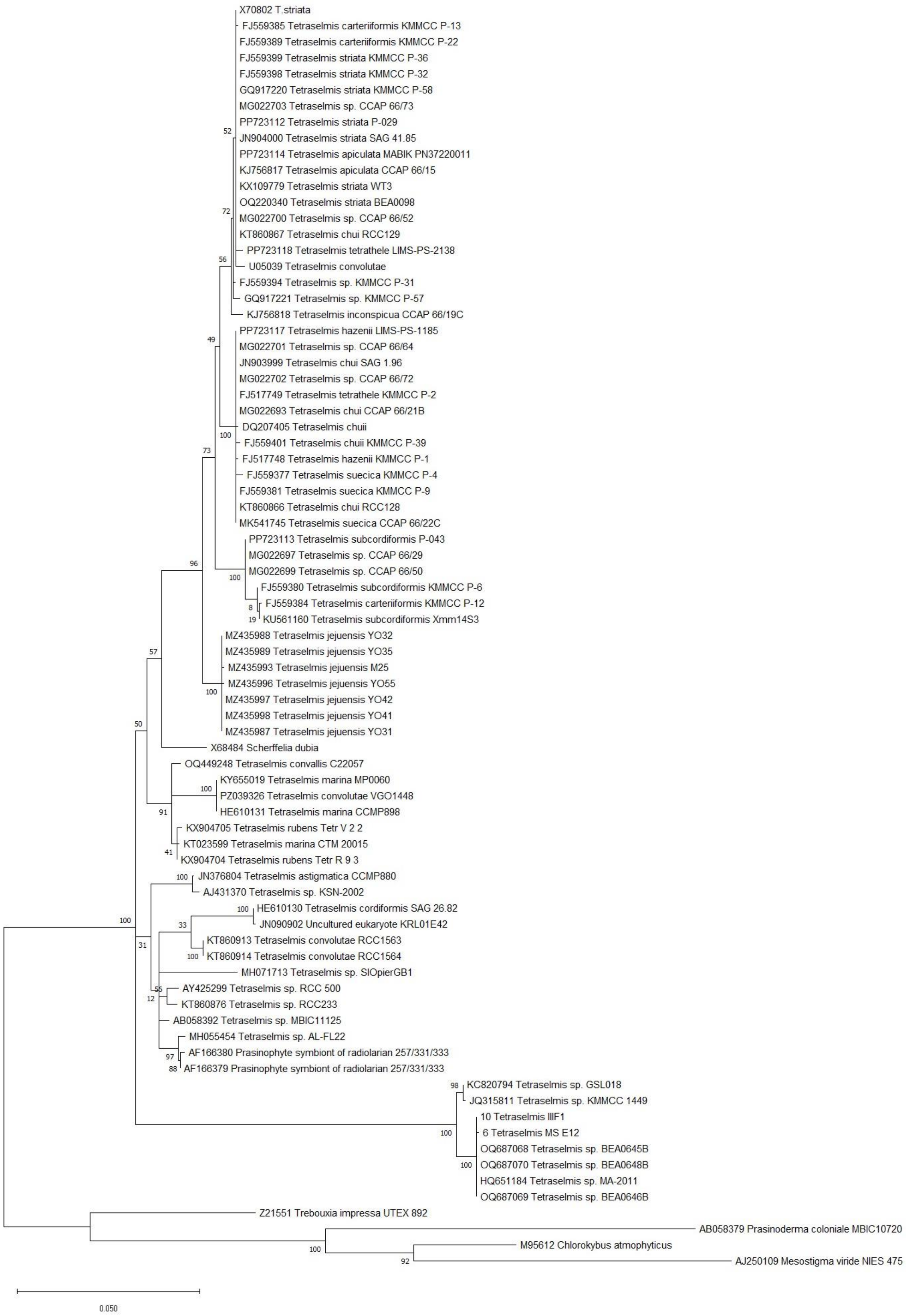

Figure S2. Maximum likelihood tree of Chlorodendrophyceae showing the phylogenetic position of the new strains ICIS64 and ICIS65, constructed based on comparison of 18S rRNA gene nucleotide sequences, constructed with MEGA11. The model Kimura-2 (G+I) was selected as the optimal evolutionary model. Statistical support for tree nodes is shown as bootstrap.

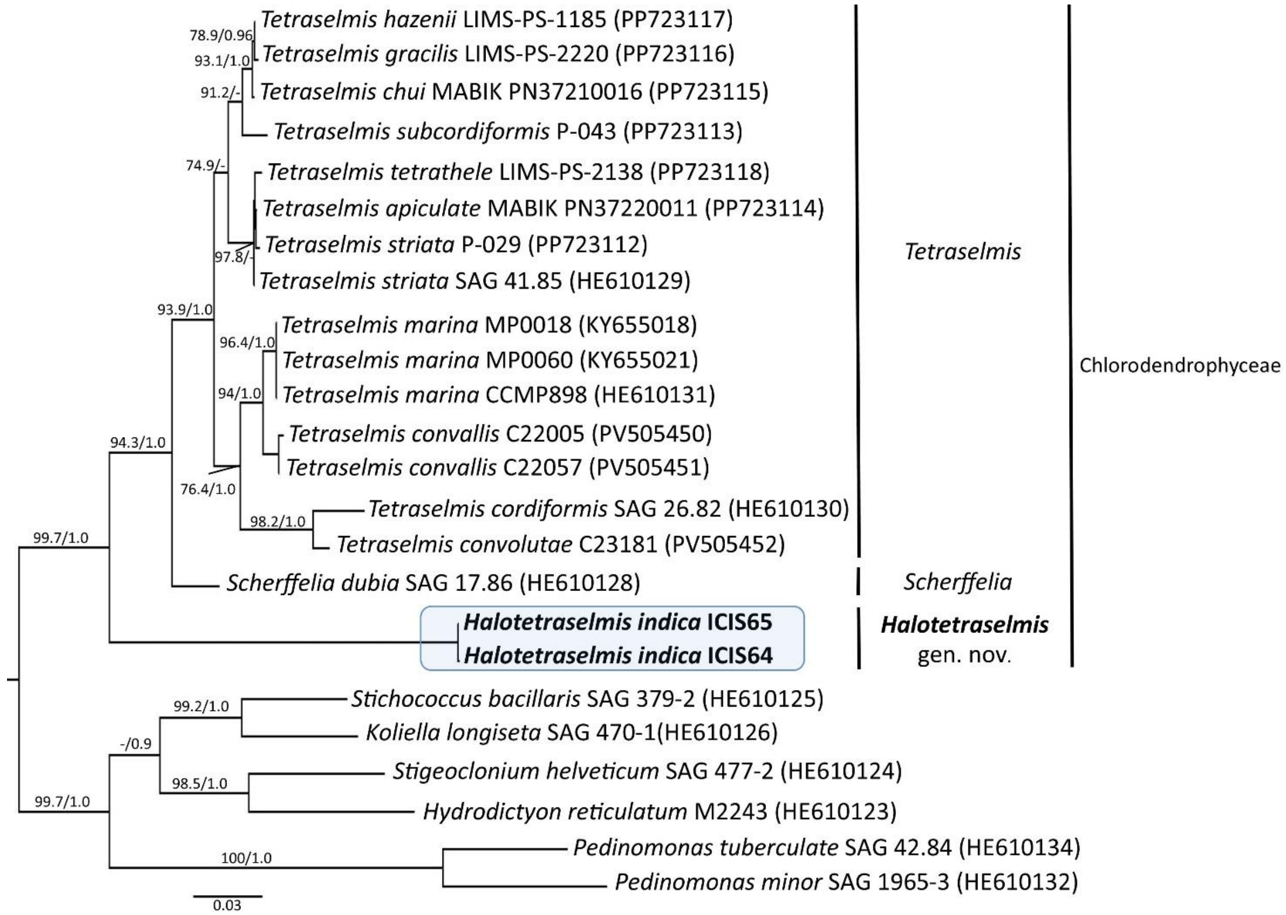

Figure S3. Maximum likelihood tree of Chlorodendrophyceae showing the phylogenetic position of the new strains ICIS64 and ICIS65, constructed based on comparison of 28S rRNA gene nucleotide sequences (1,379 bp). The TN+F+R2 model was selected as the optimal evolutionary model. Statistical support for tree nodes is shown as bootstrap and posterior probability support values (BP/PPS); BP values ≤ 70% and PPS < 0.9 are not shown. The nucleotide sequences of *Pedinomonas minor SAG 1965-3* (*HE610132*), *Pedinomonas tuberculate* SAG 42.84 (HE610134), *Hydrodictyon reticulatum* M2243 (HE610123), *Stigeoclonium helveticum* SAG 477-2 (HE610124), *Koliella longiseta* SAG 470-1 (HE610126) and *Stichococcus bacillaris SAG 379-2* (HE610125) were used as the outgroup. The strains studied are shown in bold.

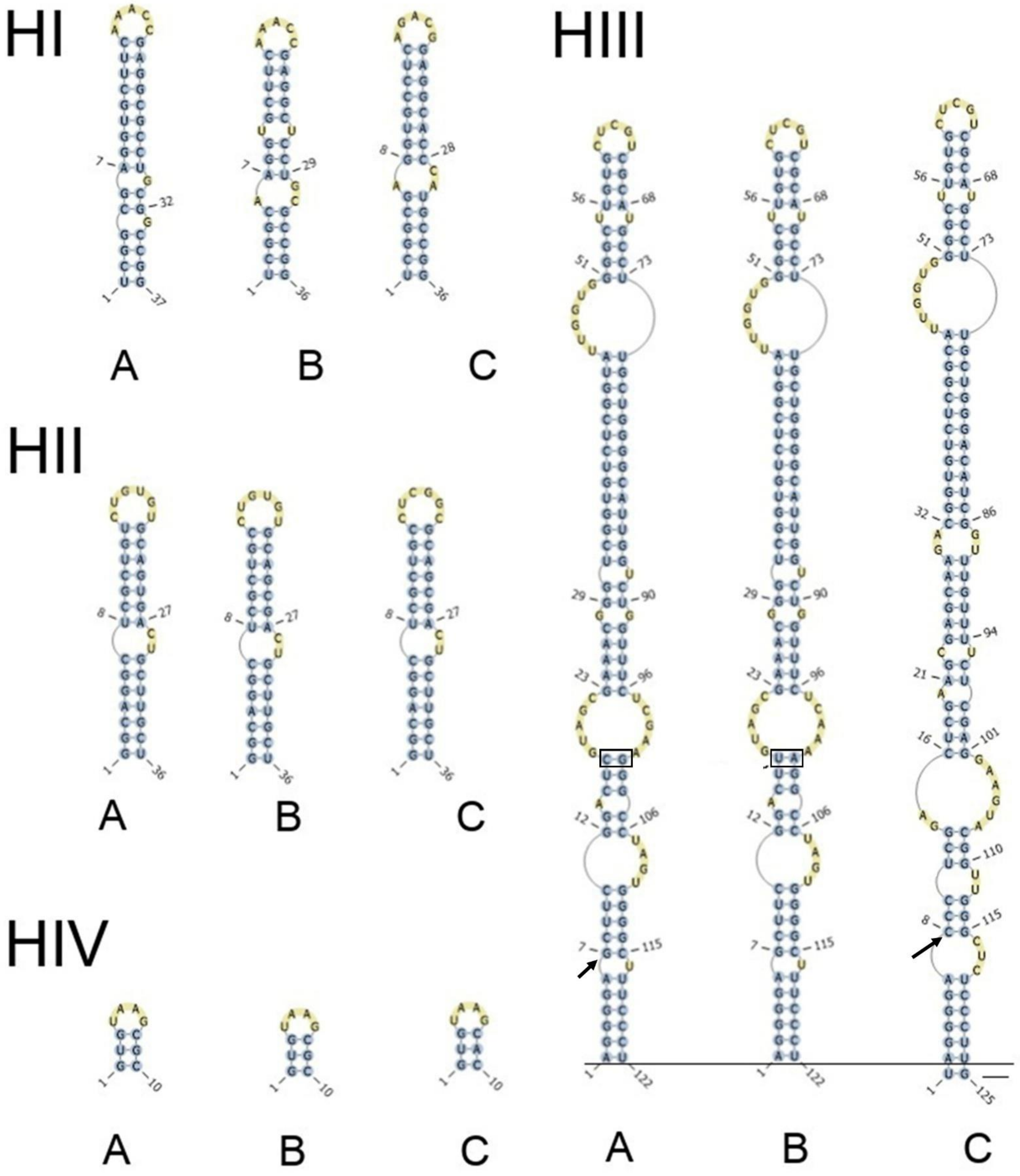

Figure S4. Differences of ITS2 secondary structure between *Halotetraselmis indica* comb. nov. and their closest homologues. A - *Halotetraselmis indica*, B - «*Tetraselmis*» sp. A5 (KU682841), C – «*Tetraselmis*» sp. KMMCC 1449 (JQ315811). CBC is highlighted with a black rectangle, inversion (CBC-like substitution) – by black arrows.

Table S1. List of nucleotide sequences used in phylogenetic analysis of Chlorodendrophyceae.

Species name	Strain No.	Synonym	Origin	GenBank sequence accession number			
				18S	ITS1-5.8S-ITS2	rbcL	28S
Tetraselmis sp.	BEA0648B		Spain: Canary Islands, Gran Canaria, Las Palmas province, La Charca de Maspalomas (seasonal brackish lagoon), Maspalomas Dunes	OQ687070			
	BEA0645B			OQ687068			
	BEA0646B			OQ687069			
Tetraselmis sp.	GSL018		USA: Utah, Great Salt Lake, evaporation pond	KC820794			
Tetraselmis indica	MA-2011		India: salterns	HQ651184			
Tetraselmis cordiformis	SAG 26.82			HE610130			HE610130
Tetraselmis marina	MP0060		South Korea: seawater	KY655019			KY655021
Tetraselmis marina	CCMP898		USA: Partridge Point, Whidbey Island, North Pacific	HE610131			HE610131
Tetraselmis sp.	KMMCC 1449		Seawater	JQ315811		JQ315496	
Uncultured eukaryote	KRL01E42		Lake Karla	JN090902			
Tetraselmis sp.	RCC 500		oceanic and coastal marine ecosystems	AY425299			
Tetraselmis sp.	MBIC11125			AB058392			
Prasinophyte symbiont of radiolarian	257/331/333		cf. *Spongodrymus* 331	AF166380			
Prasinophyte symbiont of radiolarian	257/331/333		cf. *Spongodrymus* 257	AF166379			
Tetraselmis astigmatica	CCMP880			JN376804			
Tetraselmis convolutae	VGO1448		Spain: Carnota	PZ039326			
Tetraselmis convolutae	C23181		Japan: Ishigaki (convolutid acoel (Convolutidae))	PV505174			PV505452
Tetraselmis hazenii	LIMS-PS-1185			PP723117		PP734130	PP723117
Tetraselmis sp.	CCAP 66/64		Venezuela (Marine Environment)	MG022701			
Tetraselmis chui	SAG 1.96		Antarctica: Princess Elizabeth Land, Vestfold Hills, Ekho Lake	JN903999			
Tetraselmis sp.	CCAP 66/72		USA: Washington, San Juan Island (Marine Environment)	MG022702			
Tetraselmis tetrathele	KMMCC P-2		marine	FJ517749			

Tetraselmis chui	CCAP 66/21B		UK: England, Withernsea, Yorkshire (Marine Environment)	MG022693			
Tetraselmis hazenii	KMMCC P-1/ UTEX 171	*Platymonas subcordiformis*	USA: New Haven, Connecticut (Marine Environment)	FJ517748			
Tetraselmis convolutae	RCC1563		Atlantic Ocean: English Channel, Britanny coast	KT860913			
Tetraselmis sp.	CCAP 66/52		USA: Narragansett, Rhode Island (Marine Environment)	MG022700			
Tetraselmis subcordiformis	P-043	*Platymonas subcordiformis*		PP723113		PP734126	PP723113
Tetraselmis sp.	CCAP 66/29		Monaco: Marine Environment	MG022697			
Tetraselmis striata	BEA0098		Spain: Las Palmas, Isla de Lobos, Natural Park (marine water)	OQ220340			
Tetraselmis striata	WT3		North-Eastern Tunisia: sea water	KX109779			
Tetraselmis apiculata	CCAP 66/15		UK (brackish)	KJ756817			
Tetraselmis apiculata	MABIK PN37220011		Южной Корее: Marine Environment	PP723114		PP734127	PP723114
Tetraselmis striata	SAG 41.85		United Kingdom: Conwy, North Wales (Marine Environment)	JN904000			HE610129
Tetraselmis striata	P-029			PP723112			PP723112
Tetraselmis sp.	CCAP 66/73		Japan	MG022703			
Tetraselmis suecica	KMMCC P-9		South Korea: Deukryang Bay (sea water)	FJ559381			
Tetraselmis inconspicua	CCAP 66/19C			KJ756818			
Tetraselmis sp.	CCAP 66/50		USA: Marine Environment	MG022699			
Tetraselmis tetrathele	LIMS-PS-2138			PP723118		PP734131	PP723118
Tetraselmis sp.	RCC233		Italy: Mediterranean Sea	KT860876			
Tetraselmis jejuensis	YO 3-1		South Korea: Jeju Island, supralittoral tide pools	MZ435987			
Tetraselmis jejuensis	YO 3-2			MZ435988			
Tetraselmis jejuensis	YO 3-5			MZ435989			
Tetraselmis jejuensis	YO 4-1			MZ435998			
Tetraselmis jejuensis	YO 4-2			MZ435997			
Tetraselmis verrucosa	CCAP 66/6		United Kingdom: Norfolk, England (brackish; salt marsh)	KX904700			
Tetraselmis verrucosa	Mes 17		Greece: Messolonghi (coastal lagoon)	KX904701			
Tetraselmis sp.	SIOpierGB1		USA: coastal Pacific site	MH071713			

Tetraselmis sp.	AL-FL22		Pacific Ocean: ocean water	MH055454			
Tetraselmis striata	KMMCC P58		South Korea: East sea, sea water	GQ917220			
Tetraselmis striata	KMMCC P62		South Korea: Yangyang, sea water	FJ559398			
Tetraselmis striata	PCC 443			X70802			
Tetraselmis carteriiformis	KMMCC P12		South Korea: sea water	FJ559384			
Tetraselmis subcordiformis	KMMCC P6		China: sea water	FJ559380			
Tetraselmis chui	MABIK PN37210016					PP734128	
Tetraselmis marina	CTM 20015		Tunisia: Beach of Petit Paris, Kelibia, Nabeul	KT023599		NC_065695	
Tetraselmis suecica	CCMP 904		United Kingdom, England: Isle of Wight, Bembridge, (North Atlantic Ocean)			DQ173247	
Tetraselmis suecica						NC_067041	
Tetraselmis sp.	RCC6047		Atlantic Ocean:France,Roscoff Astan			OZ412815	
Tetraselmis sp.	CCMP 881					KU167097	
Tetraselmis sp.	A5		Red Sea water		KU682841		
Tetraselmis marina	MP0018		South Korea: Jeju-do (seawater)	KY655016			KY655018
Tetraselmis convallis	C22005		Japan: Kabira (convolutid acoel (Convolutidae))	OQ449206			PV505450
Tetraselmis convallis	C22057		Japan: Kabira (convolutid acoel (Convolutidae))	OQ449248			PV505451
Tetraselmis gracilis	LIMS-PS-2220			PP723116		PP734129	PP723116
Tetraselmis chui	MABIK-ON3721001		South Korea: Gyeongsangnam-do	PP723115			PP723115
Tetraselmis verrucosa	Tetr_V_2_2	*Tetraselmis rubens*	Greece: Kalamas Delta, Vatatsa lagoon	KX904705			HE610128
Scherffelia dubia	SAG 17.86		United Kingdom Locality: Essex, small pond in Epping Forest (freshwater)	X68484			HE610128
**Outgroups**							

Trebouxia impressa	UTEX 892		photobiont of the lichen Physcia stellaris	Z21551			
Koliella longiseta	SAG 470.1			HE610126			HE610126
Stichococcus bacillaris	SAG 379.2			HE610125			HE610125
Pedinomonas minor	SAG 1965-3 (UTEX 1350)			HE610132			HE610132
Pedinomonas tuberculata	SAG 42.84 (UTEX 1539)			HE610134			HE610134
Hydrodictyon reticulatum	M2243			HE610123			HE610123
Stigeoclonium helveticum	SAG 477.2			FN824372			HE610124
Planctonema lauterbornii	SCCAP K-0187					KM438432	
Planctonemopsis distans	LXD91					KY283844	
